# Molecular Verification of the UK National Collection of Cultivated *Liriope* and *Ophiopogon* Plants

**DOI:** 10.3390/plants9050558

**Published:** 2020-04-27

**Authors:** Eva Masiero, Dipanwita Banik, John Abson, Paul Greene, Adrian Slater, Tiziana Sgamma

**Affiliations:** 1 Biomolecular Technology Group, Leicester School of Allied Health Science, Faculty of Health and Life Sciences, De Montfort University, Leicester LE1 9BH, UK; eva.masiero@dmu.ac.uk (E.M.); ads@dmu.ac.uk (A.S.); 2 CSIR-North East Institute of Science & Technology, Jorhat 785006, India; banikdipanwita@yahoo.com; 3 Brooksby Melton College, Asfordby Rd, Melton Mowbray LE13 0HJ, UK; johnabson42@gmail.com (J.A.); pwgreene270@btinternet.com (P.G.)

**Keywords:** Liriopogon, DNA barcode, haplotype, *rbcL*, nrITS, UK National Cultivated Plant Collection (NCCP)

## Abstract

A collection of cultivated *Liriope* and *Ophiopogon* plants was established in 1996–1998 and subsequently hosted at a horticultural college. Uncertainties about the identification of the accessions, compounded by potential errors in propagation and labelling have led to waning confidence in the identities of the plants in the collection. The potential for using DNA barcoding to determine the species identities of the accessions was investigated. The DNA barcode regions of the plastid ribulose-1,5-bisphosphate carboxylase/oxygenase large subunit gene (*rbcL*) and nuclear ribosomal internal transcribed spacer (nrITS) were amplified. DNA sequence analysis allowed the sequences of the accessions to be compared to reference sequences in public databases. A simple haplotype map of the characteristic polymorphic positions in the *rbcL* regions was used to clearly distinguish between the two genera and assign *Ophiopogon* accessions to individual species or sub-groups of species. The ITS sequence data confirmed these genus and species assignations and provided greater resolution to distinguish between closely related species. The combination of two DNA barcodes allowed most of the accessions to be assigned to individual species. This molecular verification confirmed the identity of about 70% of the accessions, with the remaining 30% demonstrating a range of mistaken identities at the species and genus levels.

## 1. Introduction

Over the years, there has been increasing recognition of the value of the genetic diversity captured in cultivated plants and the need to preserve, propagate and document it. The UK National Cultivated Plant Collection (NCCP) scheme makes a valuable contribution to the conservation of genetic diversity in cultivated plants (http://www.nccpg.com/National-Collections.aspx). The open model used in the UK allows any suitable collection to be registered with the NCCP, ranging from those held by internationally prestigious botanic gardens to the personal collections of individual enthusiasts. However, the overall value of the scheme is dependent upon the quality of the collections and the correct identification of cultivars within each collection. The identification of cultivated plants is often more difficult than of plants from the wild, due to their complex and often uncertain histories of selective breeding and hybridisation. This problem is exacerbated in ornamental plants, where the selection of unusual floral morphologies may have modified floral characteristics that are important for species assignment.

The English common name “Liriopogon” collectively refers to the plants belonging to the genera *Liriope* Lour. and *Ophiopogon* Ker Gawl [1]. They are commonly known as Aztec grass, Mondo grass, Monkey grass and Snake’s beard, amongst other common names [2]. They are widely cultivated in many parts of the world as excellent groundcover in various landscapes due to their hardiness and pest and disease resistance [3]. The UK National Plant Collections of *Ophiopogon* and *Liriope* are held at Brooksby Melton College, Leicestershire. The collection was donated to the college around 20 years ago and comprises some 80 accessions in two collections (54 *Liriope* and 34 *Ophiopogon*), collected largely during the years 1996–1998 by an individual collector. The collection has been maintained by the propagation of plants in glasshouse facilities and in outdoor plots. The need to re-evaluate the collection was recognised in the light of changes in classification and nomenclature over the past twenty years [2,4] and the concern that initial misidentification and subsequent mishandling and mis-labeling over decades of propagation could have reduced the reliability of cultivar identities [5,6,7,8]. The potential for the hybridisation of cultivars grown in close proximity, with the subsequent degradation of characteristic morphological features, is also a concern.

Both genera are perennial, short, rhizomatous or stoloniferous herbs and share many common morphological characters [2,7,9]. The leaves are mostly cauline, tufted and basal. The inflorescences are racemose or panicled, with flowers that are usually bisexual with an articulate pedicel. They comprise six free tepals and six stamens with the anthers basifixed. The ovary is 3-loculed and the style is columnar. The fruit is an early dehiscent berry. Morphologically, *Liriope* differs from *Ophiopogon* in having green leaves, flowers erect in inflorescences, a cupulate to rotate corolla, a superior ovary, filaments longer or as long as the anthers, apical poricidal anthers and blackish seeds [3,6]. *Ophiopogon* may have horizontally white striped leaves, but there are also several “black” varieties with dark purplish leaves [2,3,5]. In contrast to *Liriope*, the flowers are drooping in inflorescences, with a campanulate corolla, semi-inferior ovary, filaments shorter than the anthers, longitudinally dehiscent anthers and blue seeds. However, since many of the accessions in the collection do not flower regularly, routine confirmation of identity has relied solely on vegetative characters. Although a study by Zhou et al. (2009) [10] reported that hybridization between tetraploid *L. spicata* and diploid *Ophiopogon* may be occurring naturally in the wild, some of the *Liriope* species, like *L. spicata* var. prolifera, have no seeds after efflorescence, and they reproduce only by vegetative propagation [11]. Vegetative propagation is the most common practice for the propagation of *Liriope* and *Ophiopogon*.

There are nearly 65 species under the genus *Ophiopogon* and nearly eight species under the genus *Liriope*, distributed in tropical, subtropical and temperate regions from East Asia to South East Asia to Japan [12]. However, only three species under *Ophiopogon* (*O. jaburan* (Kunth) Loddiges, *O. japonicus* Ker Gawl. and *O. planiscapus* Nakai) and two species under *Liriope* (*L. spicata* (Thunb.) Lour. and *L. muscari* (Decne.) L.H.Bailey) were recorded in the European Garden Flora [13], while only *Ophiopogon* was recorded in the Handbook of North European Garden Plants [14]. In the US, a wider range of species were apparently found in cultivation, with a minimum of eight recorded for *Ophiopogon* (*O. clarkei, O. graminifolius*, *O. intermedius*, *O. jaburan*, *O. japonicus*, *O. kansuensis*, *O. ohwii* and *O. planiscapus*) [3,5] and six, for *Liriope* (*L. exiliflora, L. gigantea, L. minor, L. muscari, L. platyphylla and L. spicata*) [3,6]. Note, however, that the taxonomy of these genera has changed since this publication, with *O. graminifolius* now accepted as *L. graminifolia*, *O. kansuensis* as *L. kansuensis* and *O. ohwii* as *O. japonicus* [4]. *L. gigantea* and *L. platyphylla* are now regarded as synonyms for *L. muscari* [4], as is *L. exiliflora,* though this is still disputed by Fantz and colleagues [8].

The taxonomic placement of both genera has been controversial since the 18th century. However, with the advancement of molecular phylogenetic methods, the taxonomic placement and monophyletic origin of both genera has now been confirmed. During the 20th century, both the genera were treated under the tribe Ophiopogoneae, subfamily Ophiopogonoideae, family Convallariaceae [15,16,17]. Later, they were treated under the tribe Ophiopogoneae but transferred to the family Liliaceae [18,19]. The molecular phylogeny of Ophipogoneae based on *matK* and *rbcL* DNA sequences in the plastid genome revealed the monophyly of the tribe Ophipogoneae and also the monophyly of each of the genera *Ophiopogon* and *Liriope* within the tribe Ophipogoneae [20,21]. Currently, both genera belong to the tribe *Ophiopogoneae* (Endl.) Voigt, under the subfamily *Nolinoideae* Burnett, under the family *Asparagaceae* sensu APG III, 2009, under the order *Asparagales* Bromhead [22,23,24,25].

To date, there is a little evidence of DNA techniques being applied to the identification of cultivated, ornamental plants, but they could prove to be a useful method for the validation of National Plant Collections and as a tool for the wider horticultural community. Identification tests based on DNA barcoding would be much faster than traditional methods of identification that require growth to the flowering stage, in parallel with control plants. In the present study, the relevance of DNA-based tests to the identification and reclassification of *Liriope* and *Ophiopogon* species is highlighted.

## 2. Results

### 2.1. DNA Barcoding of the rbcLa Region of Liriope and Ophiopogon Accessions

**Figure 1:**
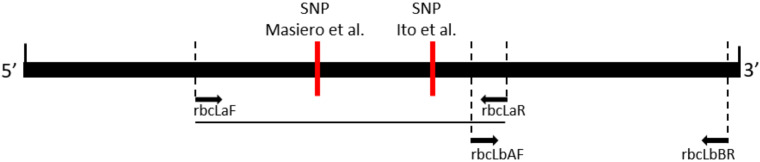
Diagram of the *rbcL* gene showing the positions of primer pairs used to amplify the central and 3′ end region of the *rbcL* barcode region. The genus-specific diagnostic single nucleotide polymorphisms (SNPs) reported by Masiero et al., (2017) [26] and Ito et al. (2015) [27] are shown in relation to the primer pairs.

**Figure 2:**
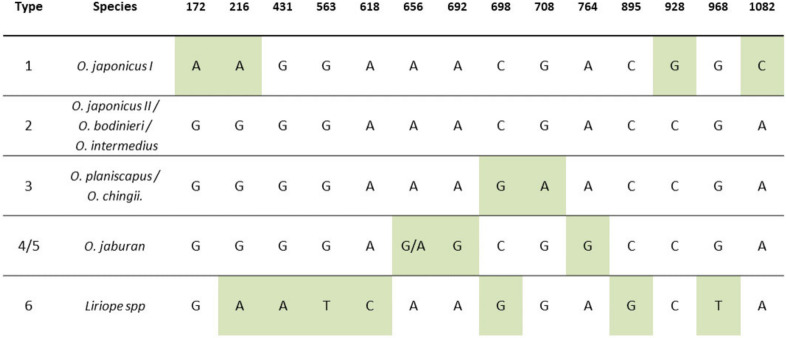
Haplotype map of the *rbcL* barcode region in different *Ophiopogon* and *Liriope* species. The map is a combination of published data from the *rbcLa* [24] and *rbcLb* [26] barcode regions, augmented with unpublished sequences from the GenBank database. Diagnostic SNPs unique to single or related groups of species are highlighted.

The ribulose bisphospate carboxylase large subunit is encoded in the plastid by the *rbcL* gene, which, in *Liriope* and *Ophiopogon*, is over 1400 bp. The DNA barcoding of the *rbcL* region typically analyses a partial sequence from either the 5′ *rbcLa* region or the 3′ *rbcLb* region (Figure 1). The sequencing of the plastid *rbcLa* barcode region of 75 of the National Collection *Ophiopogon* and *Liriope* specimens was reported previously [26]. Multiple sequence alignment of this barcode dataset along with sequences from the GenBank database revealed very little sequence variation, with just four characteristic single nucleotide polymorphic (SNP) positions observed at Positions 172, 216, 392 and 431 (Figure 2). The G/A SNP at Position 431 was found to be genus-specific, with the guanine present in *Ophiopogon* substituted by an adenine in *Liriope*. This is the target of a PCR test to distinguish the two genera (Figure 1) [26]. Four *Ophiopogon* samples (BTG_693, BTG_695, BTG_709 and BTG_711) and three *Liriope* samples (BTG_628, BTG_667 and BTG_677) were found to not fit this SNP pattern. The explanation that these accessions had been misidentified at the genus level is supported by results described in the next sections.

The remaining three SNPs at Positions 172, 216 and 392 were identical in all the confirmed *Liriope* accessions but varied between clusters of *Ophiopogon* species (Figure 2). 

### 2.2. DNA Barcoding of the rbcLb Region of Liriope and Ophiopogon

A study by Shiba et al. [28] observed a number of SNPs in the *rbcLb* region of *Liriope* and *Ophiopogon* species and identified corresponding haplotypes that were characteristic of individual species or groups of related species. A full *rbcL* haplotype map comprising 15 informative SNP positions was compiled by combining the *rbcLa* and *rbcLb* maps and retaining the haplotype categories used by Shiba et al. [28] (Figure 2). Using the Type 2 haplotype as the consensus sequence, Type 1 was defined by four SNPs (three unique); Type 3, by two SNPs (one unique); Type 4/5, by two/three unique SNPs; and Type 6 (*Liriope*), by seven SNPs (five unique) (Figure 2).

Primers were designed to amplify a region containing all the informative SNPs in the *rbcLb* region (Figure 1). This region was sequenced in all the *Ophiopogon* accessions, along with three *Liriope* specimens (BTG_628, BTG_667 and BTG_677) previously identified as having an anomalous genus-specific *rbcLa* SNP. The *rbcLb* SNP pattern for these three accessions confirmed that they all belonged in the *Ophiopogon* genus. Conversely, the detection of four misidentified *Ophiopogon* samples (BTG_693, BTG_695, BTG_709 and BTG_711) from the *rbcLa* SNP at Position 431 was confirmed by their full *rbcL* Type 6 *Liriope* haplotypes. 

The *Ophiopogon* sequences were matched with the haplotype panel, allowing the identities of those *Ophiopogon* species with a unique haplotype to be determined (Figure 2). For example, the four unique SNPs of Haplotype 1 are found only in a subgroup of *O. japonicus* (I). Accessions BTG_688, BTG_691 and BTG_692 were confirmed as *O. japonicus* (I), whilst three plants (BTG_705, BTG_706 and BTG_708) with an unknown species designation also showed this *O. japonicus* haplotype. The three samples originally designated as *O. intermedius* (BTG_682, BTG_683 and BTG_684) were also found to have a Type 1 rather than Type 2 haplotype, indicative of *O. japonicus* (I). By contrast, the accession (BTG_704) originally labelled as *O. wallichianus* (an unaccepted synonym for *O. intermedius*) had the expected Type 2 haplotype. However, the taxon originally labelled as *O. chingii* (BTG_679) showed the Type 2 rather than the expected Type 3 haplotype. 

Two samples (BTG_667 and BTG_685) were found to have the *rbcL* Haplotype 5 characteristic of *O. jaburan*; sample BTG_685 was therefore correctly designated as *O. jaburan*, but BTG_667 was originally misidentified as a *Liriope*. On the other hand, the original designation of sample BTG_686 as *O. jaburan* was not supported by the *rbcL* haplotype. One specimen, originally identified as *O. clarkei* (BTG_680), could not be identified by the *rbcL* haplotype as there are no *rbcL* sequences available in public databases. In other cases, the *rbcL* haplotype was not definitive at the species level and required supporting evidence from the internal transcribed spacer (ITS) barcoding to confirm species identity. 

### 2.3. DNA Barcoding of the nrITS Region of Liriope and Ophiopogon

Whilst *rbcL* barcoding allowed the identification of certain *Ophiopogon* species by haplotype matching, there was insufficient variation to discriminate between all species, particularly in the *Liriope* genus. In order to identify these species and resolve the apparent misidentification of certain accessions, the entire collection was subjected to DNA barcoding of the nuclear ribosomal ITS region. The sequence quality of a number of accessions was poor when sequenced in both directions using ITS1 and ITS4 primers. This was resolved in some cases by the use of the plant-specific primers ITS5P and ITS8P, but in other samples, the ITS sequence traces were of poor quality with both primer pairs, indicating low quality DNA templates. However, it was possible to resolve conflicts between the sequence traces obtained with each primer pair and by reference to other sequences in the collection, allowing these sequences to be used in the subsequent analysis.

**Figure 3:**
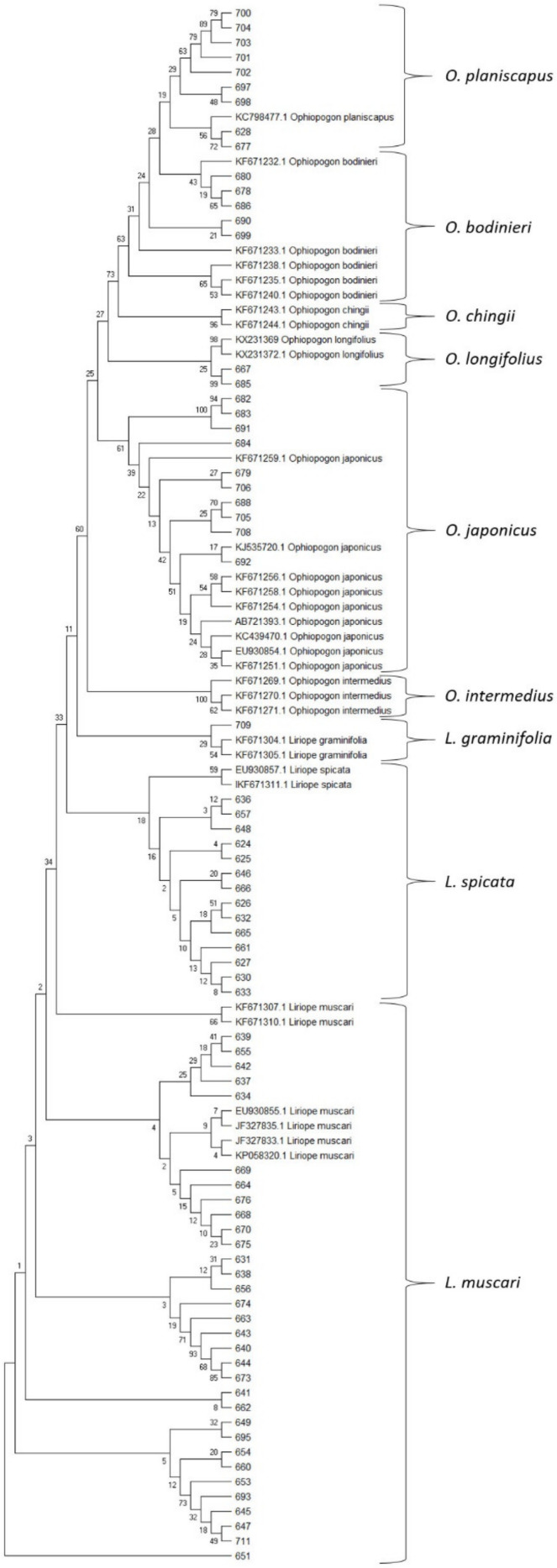
Molecular phylogenetic analysis by the Maximum Likelihood method based on ITS sequences. Ancestral states were inferred using the Maximum Likelihood method [29] and Kimura 2-parameter model [30]. The bootstrap consensus tree inferred from 1000 replicates is taken to represent the evolutionary history of the taxa analysed. Branches corresponding to partitions reproduced in less than 50% bootstrap replicates are collapsed. The percentages of replicate trees in which the associated taxa clustered together in the bootstrap test (1000 replicates) are shown next to the branches. Evolutionary analyses were conducted in MEGA X [31]. The numbers represent the BTG_ sample numbers (Table S1).

The samples’ sequenced ITS amplicons were aligned with *Liriope* and *Ophiopogon* sequences from the NCBI GenBank database. The sequences were aligned using the Clustal W MegAlign package of DNAStar (DNAStar Inc.). The evolutionary relationships of the members of both genera were inferred with the Maximum Likelihood method based on a Kimura 2-parameter model using the MEGA X software package (Figure 3). The resulting phylogenetic tree was used to identify accessions from the collection by their location on the tree (Figure 3). The tree morphology is consistent with that found by Wang et al. [4], for ITS sequences, with both *Liriope* and *Ophiopogon* supported as monophyletic genera.

All the taxa confirmed as belonging to the genus *Liriope* by *rbcL* haplotype had ITS sequences that showed close similarity to *L. muscari*, including those accessions originally identified as *L. graminifolia*, *L. minor* and *L. spicata* (Table S1). Of the five samples labelled as *L. graminifolia*, four (BTG_625, BTG_626, BTG_627 and BTG_630) clustered with *L. spicata* sequences from the database rather than with the two *L. graminifolia* sequences available in the GenBank database (KF671304.1 and KF671305.1). The fifth sample (BTG_628), labelled as *L. graminifolia*, was identified as *O. planiscapus* based on both *rbcL* and ITS sequences. 

Wang et al. [4] showed two major lineages within the genus *Ophiopogon* (Clade A and B), with *O. clarkei* and *O. intermedius* in Sub-Clade A2, *O. umbraticola* in Sub-Clade B1, *O. japonicus* in B2 and *O. bodinieri* and *O. chingii* in B3. They did not include *O. planiscapus, O. longifolius* or *O. jaburan* in their study, but the single *O. planiscapus* accession (KC798477.1) in the GenBank database showed close similarity to two *O. bodinieri* (Sub-Clade B3) accessions (KF671232.1 and KF678233.1) (Subclade B3a). The adjacent branch on the phylogenetic tree contained three further *O. bodinieri* sequences and two *O. chingii* sequences from the database (Subclade B3b). Two *O. longifolius* sequences (KX231369 and KX231372) from the database formed a distinct branch on the tree, separate from their closest relatives *O. japonicus,* and two sequences (BTG_667 and BTG_685) from the collection fell on the same branch; one of these (BTG_667) was originally classified as *L. muscari*. No *O. jaburan* ITS sequences are available in the database, but the two taxa identified as *O. jaburan* by their *rbcL* haplotype had quite distinct ITS sequences that formed a separate branch within Sub-Clade B3.

All of the *Ophiopogon* ITS sequences obtained from the plant collection fell within Sub-Clade B2 or B3. This indicates the absence of *O. intermedius* and *O. clarkei* from the collection, even though some accessions were labelled as such.

### 2.4. Deduction of Individual Taxon Identity

The combination of *rbcL* haplotype and ITS cluster analysis allowed the identity of all the taxa tested to be deduced at the sub-clade or species level. In most cases, the ITS data supported and provided further resolution to the *rbcL* results, including the seven misidentified genus taxa. In no cases was there a direct conflict between the two barcodes. 

#### 2.4.1. *Liriope* spp.

All the samples predicted by *rbcL* haplotype to belong to the genus *Liriope* had ITS sequences that confirmed this classification. The *Liriope* ITS sequences fell into two distinct groups with close similarity to either *L. muscari* or *L. spicata*. In the majority of cases, this was consistent with the original species designation. Out of 46 accessions in the Liriope collection, 27 original identifications were confirmed as correct. These 27 were all *L. muscari*. The remaining 19 misidentified taxa comprised all the plants originally designated as *L. graminifolia*, *L. minor* and *L. spicata* as well as eight of the accessions labelled as *L. muscari*. Their ITS sequences allowed the original designation to be ruled out and identification as either *L. spicata* or *L. muscari* to be supported (Table S1).

#### 2.4.2. *O. japonicus*

The identities of all the accessions showing an *O. japonicus rbcL* Type 1 or 2 haplotype were confirmed by their corresponding ITS sequences. Six accessions were originally labelled as *O. japonicus* and three of these (BTG_688, BTG_691 and BTG_692) were confirmed as correct. The other three were shown to be *O. planiscapus* (BTG_690) or *L. muscari* (BTG_693, BTG_695). Three of the undetermined *Ophiopogon* species were shown to be *O. japonicus* (BTG_705, BTG_706 and BTG_708), while the other two, BTG_709 and BTG_711, were confirmed as *Liriope spp.* The three accessions labelled as *O. intermedius* (BTG_682, BTG_683 and BTG_684) all proved to be *O. japonicus*, whilst the single accession labelled as *O. chingii* (BTG_679) was found to be the only Type II *O. japonicus* in the collection.

#### 2.4.3. *O. bodinieri* and *O. planiscapus*

The differentiation of *O. bodinieri* and *O. planiscapus* proved to be more difficult, since the two species lack resolution within the ITS sequences, so identification relied more heavily on the *rbcL* haplotype. Within the group of plants with ITS sequences related to *O. bodinieri* and *O. planiscapus*, some had the *rbcL* Type 2 haplotype (typical of *O. bodinieri* as well *as O. japonicus II*) and others had the Type 3 haplotype consistent with *O. planiscapus*. This latter sub-group included three cultivars with the “Black” phenotype (BTG_697, BTG_699 and BTG_701) characteristic of ornamental *O. planiscapus* cultivars [32] and had ITS sequences that fell within Subclade B3a as defined by Wang et al. [4]. Therefore, a combination of Type 3 *rbcL* haplotype and ITS Subclade B3a was read as support for *O. planiscapus*, whilst a Type 2 *rbcL* haplotype and ITS Subclade B3b combination was interpreted as support for *O. bodinieri*. Using this identification system, five plants originally labelled as *O. planiscapus* (BTG_697, BTG_698, BTG_700, BTG_701 and BTG_702) were confirmed as *O. planiscapus*. Two *Liriope* accessions (BTG_628 and BTG_677) were also identified as *O. planiscapus*.

Two accessions, BTG_690 and BTG_699, had anomalous barcode results because their apparent location in the ITS Subclade B3b did not match their Type 3 *rbcL* haplotype. Conversely, accessions BTG_703 and BTG_704 were located in ITS Subclade B3a, which did not match their Type 2 *rbcL* haplotype. Inspection of the Subclade B3 ITS sequences revealed three SNP positions (A/G 46, C/T 231 and C/T 545) that distinguish *O. planiscapus* (KC798477.1) from the most closely related *O. bodinieri* sequence (KF671232.1). The *O. planiscapus* SNP pattern was found in all nine of the accessions having a Type 3 *rbcL* haplotype, including BTG_690 and BTG_699. These were therefore assigned to *O. planiscapus*.

The *O. bodinieri* pattern was found in all of the accessions (BTG_678, BTG_680, BTG_686, BTG_703 and BTG_704) with a Type 2 *rbcL* haplotype. Accession BTG_678 was therefore confirmed as *O. bodinieri*, whilst the other four were re-assigned from *O. clarkeii*, *O. jaburan*, undetermined *Ophiopogon* species and *O. intermedius*, respectively.

In total, the species identities of 36 accessions were confirmed, and another four unconfirmed *Ophiopogon* accessions were identified at the species level, out of a total of 73 taxa tested. The remaining 33 taxa were misidentified, seven at the genus level and the other 26 at the species level.

## 3. Discussion

This paper describes the application of DNA barcoding to the confirmation of plant species identity in a horticultural collection of ornamental plants. The value of this molecular verification is demonstrated by the discovery of a significant proportion (46%) of mistaken identities.

There are a number of explanations for these misidentifications. One of the most notable observations is that the collection is less rich in diverse species than the original records indicated. The identification of rarer species by the original collector appears to have been over-optimistic, since all the verified species are those found in other collections in the USA and Europe [1,2,3,7] that are widely traded commercially. One possibility is that the collector was misled by new varieties with unusual features resembling species not normally found in trade. It is also not clear whether accessions were identified by vegetative characters alone, since some of the plants in the collection rarely flower.

One issue that could lead to misclassification is confusion around accepted nomenclature. This was certainly the case for accessions BTG_624 (*L. exiliflora*) and BTG_704 (*O. wallichianus*). However, in both cases, the identity inferred from DNA barcoding did not match the accepted synonym (*L. muscari* and *O. intermedius*, respectively), so the misidentifications were not just the result of changes in taxonomic nomenclature.

A more likely reason for mislabeled taxa in living collections is the gradual accumulation of errors occurring during many years of propagation and re-labelling. Over some periods, the collection was grown outdoors and prone to damage from rabbits, risking the unnoticed loss of some specimens and overgrowth of others. As mentioned above, the irregular flowering of some taxa means that the curators of the collection had to rely on leaf morphology to monitor the correct labelling of accessions.

Another compounding issue is the hybridisation of plants, either before collection or during many generations of propagation. Hybridisation between species is known to occur in *Ophiopogon* [5] and is often performed deliberately. One advantage of using a combination of plastid and nuclear barcodes is that hybridisation may be detected if there is a discrepancy between the two barcodes. However, the consistency between the *rbcL* and ITS identifications suggests that this was not a prevalent issue in this particular collection.

The use of two different barcode regions offered other benefits. The *rbcL* region allowed unambiguous differentiation between the two genera, and the haplotype panel provided a simple, unambiguous method to discriminate between four groups of *Ophiopogon* species. This provided the first stage of a “two-tier” approach to species identification [33,34]. The second stage involved determining the similarity of the ITS barcode sequence to reference barcodes using a phylogenetic tree. This gave unequivocal results for most *Liriope* and *Ophiopogon* species, where good quality reference sequences were available for comparison. The correct species designation of *O. bodinieri* and *O. planiscapus* accessions required a combination of *rbcL* haplotype and ITS SNP comparison to provide a reliable identification, given the close similarity of the ITS sequences.

The molecular testing of the collection was carried out tabula rasa, without reference to the prior labelling or morphological characteristics of the accessions. The subsequent examination of characteristic morphological features (particularly flowering) (Table S1) showed that these were consistent with the molecular classification.

One of the drivers for conducting this research was a particular interest by the curators in the “black” varieties of *Ophiopogon* (Table S1) and the fact that a number of different accessions had different cultivar names. This barcoding study was able to confirm that all of these cultivars belong to the same species, *O. planiscapus*, and there were no significant differences in their *rbcL* or ITS sequences that might support the contention that these cultivars are genetically distinct. Different “pattern based” approaches to DNA testing (DNA fingerprinting techniques), like RFLP, RAPD, ISSR or AFLP, would be more suited to detecting genetic differences between the cultivars [35,36]. These results underline the importance of not relying solely on the morphological characteristics of specimens for their identification and agree with the idea that DNA barcoding should be routinely integrated in the research of alpha taxonomists [37,38]. It is also proposed that DNA barcoding should be more widely performed as a routine quality control method in biological research and presented in publications to verify the authenticity of the samples used.

Due to the low quality of ITS sequencing results of some *Liriope* and *Ophiopogon* samples when using the conventional ITS1/4 primers, which could have been linked to fungal contamination or the poor quality of the DNA purified, some of the samples had been sequenced using the plant-specific primers (ITS5p and ITS8p) [37,39]. The use of plant-specific primers helped in improving the quality of sequencing results, allowing correct identification.

The conclusion of the study is that DNA barcoding revealed a surprising level of misidentification in a national collection of horticultural plants. This indicates that other collections of cultivated ornamental plants may be similarly unreliable as living reference standards of species identity and that DNA barcoding would be an appropriate approach to verification.

## 4. Materials and Methods

### 4.1. Plant Collections

The *Liriope* and *Ophiopogon* collections were maintained under standard glasshouse conditions, with plants of each accession cultivated in individual pots. The collection comprised one labelled pot per accession (with a few duplicates) containing single or a small number of plants. The collection records indicated a total of 54 *Liriope* accessions and 34 *Ophiopogon* accessions, but only 46 *Liriope* and 27 *Ophiopogon* taxa were available to be tested (Table S1). The Liriope collection included five named species (*L. exiliflora*, *L. graminifolia*, *L. minor*, *L. muscari* and *L. spicata*) and some taxa only identified at the genus level. The *Ophiopogon* collection included eight different species (*O. bodinieri*, *O. chingii*, *O. clarkei*, *O. intermedius*, *O. jaburan*, *O. japonicus*, *O. planiscapus* and *O. wallichianus*) along with a number of unidentified species. The *O. wallichianus* accession (BTG_704) was renamed as its accepted synonym, *O. japonicus*, and *L. exiliflora* (BTG_624) was renamed as its accepted synonym, *L. muscari* [2,5,8]. Each available accession was photographed, and these are included in the inventory of the collection shown in Table S1.

### 4.2. Plant Material and Genomic DNA Extraction

Fresh leaves were collected from 74 different accessions of *Ophiopogon* and *Liriope* at Brooksby Melton College (Melton Mowbray, Leicestershire, UK) from the UK National Plant Collections for *Ophiopogon* and *Liriope*. Details of the genera, species and accession numbers are in Table S1. Samples were stored at −80 °C. DNA was extracted from 100 mg of frozen material, previously ground to a fine powder in liquid nitrogen with a mortar and pestle, using the DNeasy Plant Mini Kit (Qiagen Inc., Germantown, MD, USA) following the manufacturers’ guidelines. DNA concentration and quality were assessed using NanoDrop (Thermofisher) technology (Table S2).

### 4.3. PCR Protocols

PCRs were carried out using primers as detailed in Table 1.

PCR reaction mixes contained 1X MyTaq Red Mix (Bioline), 0.2 μM of each forward and reverse primer, and 1 μL of gDNA as the template. A G-Storm GS1 Thermal Cycler (G-Storm Ltd., Somerton, UK) was used with the following program:

- *rbcLa* PCR: initial denaturation step of 5 min at 95 °C followed by 35 cycles consisting of 30 s at 95 °C, 20 s at 52 °C, and 50 s at 72 °C, with a final extension period of 5 min at 72 °C.
- *rbcLb* PCR: initial denaturation step of 5 min at 94 °C followed by 35 cycles consisting of 30 s at 94 °C, 30 s at 56 °C, and 30 s at 72 °C, with a final extension period of 5 min at 72 °C.
- ITS PCR: initial denaturation step of 5 min at 95 °C followed by 30 cycles consisting of 30 s at 95 °C, 30 s at 61 °C, and 30 s at 72 °C, with a final extension period of 5 min at 72 °C.
- ITSP PCR: initial denaturation step of 5 min at 95 °C followed by 30 cycles consisting of 30 s at 95 °C, 30 s at 61 °C, and 30 s at 72 °C, with a final extension period of 5 min at 72 °C.

PCR products were run on 2% (w/v) agarose, 1X TBE gels with 1 μL of SYBR^®^ Safe DNA Gel Stain (Invitrogen, Paisley, United Kingdom) at 100 V for 30 min and analysed in a Gel Doc™ EZ Gel Documentation System (BioRad, Oxford, United Kingdom). PCR products were submitted for sequence analysis to Macrogen (www.dna.macrogen-europe.com) to verify the authenticity of the starting material.

### 4.4. DNA Sequence Analysis

The obtained sequences were used to implement contigs and generate multiple sequence alignments using the CLC Main Workbench 7.5.1 software (Qiagen, Germantown, MD, USA) (Supplementary Data S1). To create the alignment, the following parameters were selected: Gap open cost = 10.0, Gap extension cost = 1.0, and the very accurate progressive alignment was selected.

Published *Liriope* and *Ophiopogon* ITS DNA sequences were obtained from the National Center for Biotechnology Information (NCBI) GenBank database (http://www.ncbi.nlm.nih.gov/).

Phylogenetic analyses were conducted using the MEGA X [29] software package. The evolutionary history was inferred with the Maximum Likelihood method [30] based on the Kimura 2-parameter model [31]. 

## Figures and Tables

**Table 1:** List of primers, with relative Ta and predicted band size.

Primers	References	Sequences 5′–3′	Annealing Temperature	Amplicon Size (bp)
rbcLaF	[40]	ATGTCACCACAAACAGAGACTAAAGC	52 °C	≃ 500/600
rbcLaR	[41]	GTAAAATCAAGTCCACCRCG		
rbcLbAF	[28]	CGGTGGACTTGATTTTACCA	56 °C	≃ 500
rbcLbBR		TCATCACGTAATAAATCAAC		
ITS1	[42]	TCCGTAGGTGAACCTGCGG	61 °C	≃ 600
ITS4		TCCTCCGCTTATTGATATGC		
ITS5P ITS8P	[37]	GGAAGGAGAAGTCGTAACAAGG CACGCTTCTCCAGACTACA	61 °C	≃ 600

## Supplementary Materials

The following are available online at https://www.mdpi.com/2223-7747/9/5/558/s1, Table S1: Liriope and Ophiopogon samples’ list and their identification, Supplementary Data S1: *Liriope* and *Ophiopogon* samples ITS DNA sequences.

## Abbreviations

**nrITS**	nuclear ribosomal internal transcribed spacer
**rbcL**	large subunit of ribulose-1,5-bisphosphate carboxylase/oxygenase gene
