# Supplementary material for: Molecular Verification of the UK National Collection of Cultivated Liriope and Ophiopogon Plants
Source: Plants (Basel). 2020 Apr 27;9(5):558. doi: 10.3390/plants9050558 (PMC7284512; doi:10.3390/plants9050558)
Supplement: Supplementary file 1 [file plants-09-00558-s001.zip › Supplemental Data 1.docx]

Supplemental Data 1: *Liriope* and *Ophiopogon* samples ITS DNA sequences

>ITS_EU930857.1_Liriope_s

CGAACCCGTAAACGCTCCCGCAGGGGCGGAGGGAGGGCGGATATTCCGACCGCCCGACCC

CGCACCTCGGGGCACAATGGCCGCCCCCGCCCCGCGTCGCTGCGGGACGGGCGGCGGGAA

CAACACCCGGCGCGGTGGGCGCCAAGGAACAGTGCTGTCGGAGAGCGCGCCGCGCAGCGC

GGTCTTTCCATACGTCGAACTTTTACGACTCTCGGCAACGGATATCTTGGCTCTCGCATC

GATGAAGAACGTAGCGAAATGCGATACTTGGTGTGAATTGCAGAATCCCGTGAACCATCG

AGTCTTTGAACGCAAGTTGCGCCCGAGGCTATCCGGCCGAGGGCACGCCTGCCTGGGCGT

CACGCCTCGCGTCGCTCCGCGCACCCTGCCCCCCTTCCCGGGGAGGCGGCGGGCGCAGAT

GCGGAGATTGGCCCCCCGTGCATGACGGCGCGGCGGGTCGAAGTGCGTGCCGCCGGCCGG

GACGGACGCGGCGAGTGGTGGACGGACACGTGCGGCGCTCAACGCCGCCTCCGCCCCCGG

TCCCGGAGGTGCATGCAAGGAACCCACGCCGAGCGCCCCTCGGAACACGACCCCAGGTCA

G

>ITS_KF671311.1_Liriope_s

CGAACCCGTAAACGCTCCCGCAGGGGCGGAGGGAGGGCGGATATTCCGACCGCCCGACCC

CGCACCTCGGGGCACAATGGCCGCCCCCGCCCCGCGTCGCTGCGGGACGGGCGGCGGGAA

CAACACCCGGCGCGGTGGGCGCCAAGGAACAGTGCTGTCGGAGAGCGCGCCGCGCAGCGC

GGTCTTTCCATACGTCGAACTTTTACGACTCTCGGCAACGGATATCTTGGCTCTCGCATC

GATGAAGAACGCAGCGAAATGCGATACTTGGTGTGAATTGCAGAATCCCGTGAACCATCG

AGTCTTTGAACGCAAGTTGCGCCCGAGGCTATCCGGCCGAGGGCACGCCTGCCTGGGCGT

CACGCCTCGCGTCGCTCCGCGCACCCTGCCCCCCTTCCCGGGGAGGCGGCGGGCGCAGAT

GCGGAGATTGGCCCCCCGTGCCTGACGGCGCGGCGGGTCGAAGTGCGTGCCGCCGGCCGG

GACGGACGCGGCGAGTGGTGGACGGACACGTGCGGCGCTCAACGCCGCCTCCGCCCCCGG

TCCCGGAGGTGCATGCAAGGAACCCACGCCGAGCGCCCCTCGGAACACGACCCCAGGTCA

G

>Lir_624_1255_EM_ITS_ITS1

GGGCGGATATTCCGACCGCCCGACCCCGCACCTCGGGGCACAATGGCCGCCCCCGCCCCG

CGTCGCTGCGGGACGGGCGGCGGGAACAACACCCGGCGCGGTGGGCGCCAAGGAACAGTG

CTTTCGGAGAGCGCGCCGCGCAGCGCGGTCTTTCCATACGTCGAACTTTTACGACTCTCG

GCAACGGATATCTTGGCTCTCGCATCGATGAAGAACGTAGCGAAATGCGATACTTGGTGT

GAATTGCAGAATCCCGTGAACCATCGAGTCTTTGAACGCAAGTTGCGCCCGAGGCTATCC

GGCCGAGGGCACGCCTGCCTGGGCGTCACGCCTCGCGTCGCTCCGCGCACCCTGCCCCCC

TTCCCGGGGAGGCGGCGGGCGCAGATGCGGAGATTGGCCCCCCGTGCCTGACGGCGCGGC

GGGTCGAAGTGCGTGCCGCCGGCCGGGACGGACGCGGCGAGTGGTGGACGGACACGTGCG

GCGCTCAACGTCGCCTCCGCCCCCGGTCCCGGAGGTGCA

>Lir_625_1256_EM_ITS_ITS1

GAGGGCGGATATTCCGACCGCCCGACCCCGCACCTCGGGGCACAATGGCCGCCCCCGCCC

CGCGTCGCTGCGGGACGGGCGGCGGGAACAACACCCGGCGCGGTGGGCGCCAAGGAACAG

TGCTTTCGGAGAGCGCGCCGCGCAGCGCGGTCTTTCCATACGTCGAACTTTTACGACTCT

CGGCAACGGATATCTTGGCTCTCGCATCGATGAAGAACGTAGCGAAATGCGATACTTGGT

GTGAATTGCAGAATCCCGTGAACCATCGAGTCTTTGAACGCAAGTTGCGCCCGAGGCTAT

CCGGCCGAGGGCACGCCTGCCTGGGCGTCACGCCTCGCGTCGCTCCGCGCACCCTGCCCC

CCTTCCCGGGGAGGCGGCGGGCGCAGATGCGGAGATTGGCCCCCCGTGCCTGACGGCGCG

GCGGGTCGAAGTGCGTGCCGCCGGCCGGGACGGACGCGGCGAGTGGTGGACGGACACGTG

CGGCGCTCAACGTCGCCTCCGCCCCCGGTCCCGGAGGTGCATGCAAGGAACCCACGCCGA

>Lir_626_1257_EM_ITS_ITS1

GGGGCACAATGGCCGCCCCCGCCCCGCGTCGCTGCGGGACGGGCGGCGGGAACAACACCC

GGCGCGGTGGGCGCCAAGGAACAGTGCTTTCGGAGAGCGCGCCGCGCAGCGCGGTCTTTC

CATACGTCGAACTTTTACGACTCTCGGCAACGGATATCTTGGCTCTCGCATCGATGAAGA

ACGTAGCGAAATGCGATACTTGGTGTGAATTGCAGAATCCCGTGAACCATCGAGTCTTTG

AACGCAAGTTGCGCCCGAGGCTATCCGGCCGAGGGCACGCCTGCCTGGGCGTCACGCCTC

GCGTCGCTCCGCGCACCCTGCCCCCCTTCCCGGGGAGGCGGCGGGCGCAGATGCGGAGAT

TGGCCCCCCGTGCCTGACGGCGCGGCGGGTCGAAGTGCG

>Lir_627_1258_EM_ITS_ITS1

GGGCGGATATTCCGACCGCCCGACCCCGCACCTCGGGGCACAATGGCCGCCCCCGCCCCG

CGTCGCTGCGGGACGGGCGGCGGGAACAACACCCGGCGCGGTGGGCGCCAAGGAACAGTG

CTTTCGGAGAGCGCGCCGCGCAGCGCGGTCTTTCCATACGTCGAACTTTTACGACTCTCG

GCAACGGATATCTTGGCTCTCGCATCGATGAAGAACGTAGCGAAATGCGATACTTGGTGT

GAATTGCAGAATCCCGTGAACCATCGAGTCTTTGAACGCAAGTTGCGCCCGAGGCTATCC

GGCCGAGGGCACGCCTGCCTGGGCGTCACGCCTCGCGTCGCTCCGCGCACCCTGCCCCCC

TTCCCGGGGAGGCGGCGGGCGCAGATGCGGAGATTGGCCCCCCGTGCCTGACGGCGCGGC

GGGTCGAAGTGCGTGCCGCCGACCGGGACGGACGCGGCGAGTGGTGGACGGACACGTGCG

GCGCTCAACGTCGCCTCCGCCCCCGGTCCCGGAGGTGCATGCAAGGAACCCACGCCGAGC

GCCCCTCGGAACACGACCCCAGGTCAG

>Lir_630_1260_EM_ITS_ITS1

GCGGATATTCCGACCGCCCGACCCCGCACCTCGGGGCACAATGGCCGCCCCCGCCCCGCG

TCGCTGCGGGACGGGCGGCGGGAACAACACCCGGCGCGGTGGGCGCCAAGGAACAGTGCT

TTCGGAGAGCGCGCCGCGCAGCGCGGTCTTTCCATACGTCGAACTTTTACGACTCTCGGC

AACGGATATCTTGGCTCTCGCATCGATGAAGAACGTAGCGAAATGCGATACTTGGTGTGA

ATTGCAGAATCCCGTGAACCATCGAGTCTTTGAACGCAAGTTGCGCCCGAGGCTATCCGG

CCGAGGGCACGCCTGCCTGGGCGTCACGCCTCGCGTCGCTCCGCGCACCCTGCCCCCCTT

CCCGGGGAGGCGGCGGGCGCAGATGCGGAGATTGGCCCCCCGTGCCTGACGGCGCGGCGG

GTCGAAGTGCGTGCCGCCGACCGGGACGGACGCGGCGAGTGGTGGACGGACACGTGCGGC

GCTCAACGTCGCCTCCGCCCCCGGTCCCGGAGGTGCATGCAAGGAACCCACGCCGAGCGC

CCCTCGGAACACGACCCCAGGTCAG

>Lir_632_1262_EM_ITS_ITS1

GAGGGCGGATATTCCGACCGCCCGACCCCGCACCTCGGGGCACAATGGCCGCCCCCGCCC

CGCGTCGCTGCGGGACGGGCGGCGGGAACAACACCCGGCGCGGTGGGCGCCAAGGAACAG

TGCTTTCGGAGAGCGCGCCGCGCAGCGCGGTCTTTCCATACGTCGAACTTTTACGACTCT

CGGCAACGGATATCTTGGCTCTCGCATCGATGAAGAACGTAGCGAAATGCGATACTTGGT

GTGAATTGCAGAATCCCGTGAACCATCGAGTCTTTGAACGCAAGTTGCGCCCGAGGCTAT

CCGGCCGAGGGCACGCCTGCCTGGGCGTCACGCCTCGCGTCGCTCCGCGCACCCTGCCCC

CCTTCCCGGGGAGGCGGCGGGCGCAGATGCGGAGATTGGCCCCCCGTGCCTGACGGCGCG

GCGGGTCGAAGTGCGTGCCGCCGACGGGGACGGACGCGG

>Lir_633_1263_EM_ITS_ITS1

AGGGCGGATATTCCGACCGCCCGACCCCGCACCTCGGGGCACAATGGCCGCCCCCGCCCC

GCGTCGCTGCGGGACGGGCGGCGGGAACAACACCCGGCGCGGTGGGCGCCAAGGAACAGT

GCTTTCGGAGAGCGCGCCGCGCAGCGCGGTCTTTCCATACGTCGAACTTTTACGACTCTC

GGCAACGGATATCTTGGCTCTCGCATCGATGAAGAACGTAGCGAAATGCGATACTTGGTG

TGAATTGCAGAATCCCGTGAACCATCGAGTCTTTGAACGCAAGTTGCGCCCGAGGCTATC

CGGCCGAGGGCACGCCTGCCTGGGCGTCACGCCTCGCGTCGCTCCGCGCACCCTGCCCCC

CTTCCCGGGGAGGCGGCGGGCGCAGATGCGGAGATTGGCCCCCCGTGCCTGACGGCGCGG

CGGGTCGAAGTGCGTGCCGCCGACCGGGACGGACGCGGCGAGTGGTGGACGGACACGTGC

GGCGCTCAACGTCGCCTCCGCCCCCGGTCCCGGAGGTGCA

>Lir_636_1265_EM_ITS_ITS1

GGCGGGAACAACACCCGGCGCGGTGGGCGCCAAGGAACAGTGCTTTCGGAGAGCGCGCCG

CGCAGCGCGGTCTTTCCATACGTCGAACTTTTACGACTCTCGGCAACGGATATCTTGGCT

CTCGCATCGATGAAGAACGTAGCGAAATGCGATACTTGGTGTGAATTGCAGAATCCCGTG

AACCATCGAGTCTTTGAACGCAAGTTGCGCCCGAGGCTATCCGGCCGAGGGCACGCCTGC

CTGGGCGTCACGCCTCGCGTCGCTCCGCGCACCCTGCCCCCCTTCCCGGGGAGGCGGCGG

>Lir_646_1275_EM_ITS5pM_ITS5p

GCGGATATTCCGACGGCCCGACCCCGCACCTCGGCGTTTAATGGCCGCCCCCGCCCCGCG

TCGCTCCGGGACGGGCGGCGGGAACAACACCCGGCGGGGTGGGCGCCAAGGAACAGTGCT

TTCGGAGAGCGCGCCGCGCAGCGCGGTCTTTCCATACCTCTAATTTTTACCACTCTAGTT

TACGGACATTTGGGCTCTGACATCTATGAACAACGTATCGTAATGAAATACTTGGTGTGA

ATTGCATAATCCCGTGTACCAACGAGTCTTTGAACGCAAGTCGCGCCCGAGGCTATCCGG

CCGAGGGCACGCCAGCCAGGGCGTCACGCCTCGCGTCTCTCCGCGCGCCCTGCCCCCCTT

CCCGGGGAGGCGGCGGGCGCAGATACGGAGATTGGACCCCCGTGCCTGACGGCGCGGCGG

GTCGAACTGCGTGCCGCCG

>Lir_648T_1277_EM_ITS_ITS1

CGAACCCGTAAACGCTCCCGCAGGGGCGGAGGGAGGGCGGATATTCCGACCGCCCGACCC

CGCACCTCGGGGCACAATGGCCGCCCCCGCCCCGCGTCGCTGCGGGACGGGCGGCGGGAA

CAACACCCGGCGCGGTGGGCGCCAAGGAACAGTGCTTTCGGAGAGCGCGCCGCGCAGCGC

GGTCTTTCCATACGTCGAACTTTTACGACTCTCGGCAACGGATATCTTGGCTCTCGCATC

GATGAAGAACGTAGCGAAATGCGATACTTGGTGTGAATTGCAGAATCCCGTGAACCATCG

AGTCTTTGAACGCAAGTTGCGCCCGAGGCTATCCGGCCGAGGGCACGCCTGCCTGGGCGT

CACGCCTCGCGTCGCTCCGCGCACCCTGCCCCCCTTCCCGGGGAGGCGGCGGGCGCAGAT

GCGGAGATTGGCCCCCCGTGCCTGACGGCGCGGCGGGTCCAAGTGCGTGCCGCCGGCCGG

GACGGACGCGGCGAGTGGTGGACGGACACGTGCGGCGCTCAACGTCGCCTCCGCCCCCGG

TCCCGGAGGTGCATGCAAGGAACCCACGCCGAGCGCCCCTCGGAACACGACCCCAGGTCA

G

>Lir_657_1327_EM_ITS5p_ITS5p-1

AAACGCTCCCGCAGGGGCGGAGGGAGGGCGGATATTCCGACCGCCCGACCCCGCACCTCG

GGGCACAATGGCCGCCCCCGCCCCGCGTCGCTGCGGGACGGGCGGCGGGAACAACACCCG

GCGCGGTGGGCGCCAAGGAACAGTGCTTTCGGAGAGCGCGCCGCGCAGCGCGGTCTTTCC

ATACGTCGAACTTTTACGACTCTCGGCAACGGATATCTTGGCTCTCGCATCGATGAAGAA

CGTAGCGAAATGCGATACTTGGTGTGAATTGCAGAATCCCGTGAACCATCGAGTCTTTGA

ACGCAAGTTGCGCCCGAGGCTATCCGGCCGAGGGCACGCCTGCCTGGGCGTCACGCCTCG

CGTCGCTCCGCGCACCCTGCCCCCCTTCCCGGGGAGGCGGCGGGCGCAAATGCGGAGATT

GGCCCCCCGTGCCTGACGGCGCGGCGGGTCGAAGTGCGTGCCGCCGGCCGGGACGGACGC

GGCGAGTGGTGGACGGACACGTGCGGCGCTCAACGTCGCC

>Lir_661_1329_EM_ITS_ITS1

GGCCGCCCCCGCCCCGCGTCGCTGCGGGACGGGCGGCGGGAACAACACCCGGCGCGGTGG

GCGCCAAGGAACAGTGCTTTCGGAGAGCGCGCCGCGCAGCGCGGTCTTTCCATACGTCGA

ACTTTTACGACTCTCGGCAACGGATATCTTGGCTCTCGCATCGATGAAGAACGTAGCGAA

ATGCGATACTTGGTGTGAATTGCAGAATCCCGTGAACCATCGAGTCTTTGAACGCAAGTT

GCGCCCGAGGCTATCCGGCCGAGGGCACGCCTGCCTGGGCGTCACGCCTCGCGTCGCTCC

GCGCACCCTGCCCCCCTTCCCGGGGAGGCGGCGGGCGCAGATGCGGAGATTGGCCCCCCG

TGCCTGACGGCGCGGCGGGTCGAAGTGCGTGCCGCCGACCGGGACGGACGCGGCGAGTGG

TGGACGGACACGTGCGGCGCTCAACGTCGCCTCCGCCCC

>Lir_665_1333_EM_ITS_ITS1

CGTCGCTGCGGGACGGGCGGCGGGAACAACACCCGGCGCGGTGGGCGCCAAGGAACAGTG

CTTTCGGAGAGCGCGCCGCGCAGCGCGGTCTTTCSATACGTCBAACTTTTACGACTCTCG

GCAACGGATATCTTGGCTCTCGCATCGATGAAGAACGTAGCGAAATGCGATACTTGGTGT

GAATTGCAGAATCCCGTGAACCATCGAGTCTTTGAACGCAAGTTGCGCCCGAGGCTATCC

GGCCGAGGGCACGCCTGCCTGGGCGTCACGCCTCGCGTCGCTCCGCGCACCCTGCCCCCC

TTCCCGGGGAGGCGGCGGGCGCAGATGCGGAGATTGGCCCCCCGTGCCTGACGGCGCGGC

GGGTCGAAGTGCGTGCCGCCGGCCGGGACGGACGCGGCAAGTGGTGGACGGACACGTGYG

GCGCTCAACGTCGCCTCCGCCCCCGGTCCCGGAGGTGCAT

>lir_666_1334_EM_ITS_ITS1

AATGGCCGCCCCCGCCCCGCGTCGCTKCGGGACGGGCGGCGGGAACAACACCCGGCGCGG

TGGGCGCCAAGGAACAGTGCTTTCGGAGAGCGCGCCGCGCAGCGCGGTCTTTCCATACGT

CGAABTTTTACGACTCTCGGCAACGGATATCTTGGCTCTCGCATCGATKAAGAACGTAGC

GAAATGCGATACTTGGTGTGAATTGCAGAATCCCGTGAACCATCGAGTCTTTGAACGCAA

GTTGCGCCCGAGGCTATCCGGCCGAGGGCACGCCTGCCTGGGCGTCACGCCTCGCGTCGC

TCCGCGCACCCTGCCCCCCTTCCCGGGGAGGCGGCGGGCGCAGATGCGGARATTGGCCCC

CCGTGCCTGACGGCGCGGCGGGTCGAAGTGCGTGCCGCCGGCCGGGACGGACGCGGCGAG

TGGTGGACGGACACGTGCGGCGCTCAACGTCGCCTCCGCCCCCGGTCCCGGAGGTGCAT

>ITS_KF671312.1_Liriope_s

GCATCGATGAAGAACGCAGCGAAATGCGATACTTGGTGTGAATTGCAGAATCCCGTGAAC

CATCGAGTCTTTGAACGCAAGTTGCGCCCGAGGCTATCCGGCCGAGGGCACGCCTGCCTG

GGCGTCACGCCTCGCGTCGCTCCGCGCTCCCTGCCCCCCTTCCCGGGGAGGCGGCGGGCG

CAGATGCGGAGATTGGCCCCCCGTGCCTGTCGGCGCGGCGGGTCGAAGTGCGTGCCGCCG

GCCGGGACGGACGCGGCGAGTGGTGGACGGACACGTGCGGCGCTCAACGTCGCCTCCGCC

CCCGGTCCCGGAGGTGCAAGCAAGGAACCCACGCCGAGCGCCCCTCGGAACACGACCCCA

GGTCAG

>ITS_KF671310.1__Liriope_m

CGAACCCGTAAACGCTCCCGCAGGGGCGGAGGGAGGGCGGATATTCCGACCGCCCGACCC

CGCACCTCGGGGCACAATGGCCGCCCCCGCCCCGCATCGCTGCGGGACGGGCGGCGGGAA

CAACACCCGGCGCGGCGGGCGCCAAGGAACAGTGCTGTCGGAGATCGCCGCGCGCCGGCC

TCGGCGCGCAGCGCGGTCTTTCCATACGTCGAACTTTTACGACTCTCGGCAACGGATATC

TTGGCTCTCGCATCGATGAAGAACGTAGCGAAATGCGATACTTGGTGTGAATTGCAGAAT

CCCGTGAACCATCGAGTCTTTGAACGCAAGTTGCGCCCGAGGCTATCCGGCCGAGGGCAC

GCCTGCCTGGGCGTCACGCCTCGCGTCGCTCCGCGCACCCTCCCCCCGTCCCGGGGAGGC

GGCGGGCGCAGATGCGGAGATTGGCCCCCCGTGCCTCACGGCGCGGCGGGTCGAAGTGCG

TGCCGCCGGCCGGGACGGACGCGGCGAGTGGTGGACGGACACGTGCGGCGCTCAACGTCG

CATCCGCCCCCCGGCCCCGGAGGTGCATGCAAGGAACCCACGCCGAGTGCCCCTCGGAAC

ACGACCCCAGGTCAG

>ITS_JF327835.1_Liriope_m

GAAGCAGTAGAAGCTCCTCCAGCGGGGGAGGGAGGGGGGATAGTATGTCCGCCCGACCCC

GCACCTCGGGGCACAACGGCCGCCCCCGCCCCGCATCGCTGCGGGACGGGCGGCGGGAAC

AACACCCGGCGCGGTGGGCGCCAAGGAACAGTGCTGTCGGAGACCGCCGCGCGCCGGCCT

CGGCGCGCGGCGCGGTCTTTCCATACGTCGAACTTTTAAGACTTTCGGCAACGGATATCT

TGGCTCTCGCATCGATGAAGAACGTAGAGAAATGCGATACTTGGTGTGAATTGCAGAATC

CCGTGAACCATGGAGTCTTTGAACGCAAGTTGCGCCCGAGGCTATCCGGCCGAGGGCACG

CCTCCCTGGGCGTCACGCCTCGCGTCGCTCCGCGCACCCTGCCCCCCGTCCCGGGGAGGC

GGCGGGCGCAGATGCGGAGATTGGCCCCCCGTGCCTCACGGCGCGGCGGGCCGAAGTGCG

TGCCGCCGGCCGGGACGGACGCGGCGAGTGGTGGACGGACACGTGCGGCGCTCAACGTCG

CCTCCGCCCCCCGGCCCCGGAGGTGCATGCAAGGAACCCACGCCGAGCGCCCCTCGGAAC

A

>ITS_KF671307.1_Liriope_m

CGAACCCGTAAACGCTTCCGCAGGGGCGGAGGGAGGGCGGATATTCCGACCGCCCGACCC

CGCACCTCGGGGCACAATGGCCGCCCCCGCCCCGCATCGCTGCGGGACGGGCGGCGGGAA

CAACACCCGGCGCGGCGGGCGCCAAGGAACAGTGCTGTCGGAGACCGCCGCGCGCCGGCC

TCGGCGCGTAGCGCGGCCTTTCCATACGTCGAACTTTTACGACTCTCGGCAACGGATATC

TTGGCTCTCGCATCGATGAAGAACGTAGCGAAATGCGATACTTGGTGTGAATTGCAGAAT

CCCGTGAACCATCGAGTCTTTGAACGCAAGTTGCGCCCGAGGCTATCCGGCCGAGGGCAC

GCCTGCCTGGGCGTCACGCCTCGCGTCGCTCCGCGCACCCTCCCCCCGTCCCGGGGAGGC

GGCGGGCGCAGATGCGGAGATTGGCCCCCCGTGCCTCACGGCGCGGCGGGTCGAAGTGCG

TGCCGCCGGCCGGGACGGACGCGGCGAGTGGTGGACGGACACGTGCGGCGCTCAACGTCG

CATCCGCCCCCGGCCCCGGAGGTGCATGCAAGGAACCCACGCCGAGCGCCCCTCGGAACA

CGACCCCAGGTCAG

>ITS_EU930855.1_Liriope_m

CGAACCCGTAAACGCTCCCGCAGGGGCGGAGGGAGGGCGGATATTCCGACCGCCCGACCC

CGCACCTCGGGGCACAACGGCCGCCCCCGCCCCGCATCGCTGCGGGACGGGCGGCGGGAA

CAACACCCGGCGCGGTGGGCGCCAAGGAACAGTGCTGTCGGAGACCGCCGCGCGCCGGCC

TCGGCGCGTGGCGCGGTCTTTCCATACGTCGAACTTTTACGACTCTCGGCAACGGATATC

TTGGCTCTCGCATCGATGAAGAACGTAGCGAAATGCGATACTTGGTGTGAATTGCAGAAT

CCCGTGAACCATCGAGTCTTTGAACGCAAGTTGCGCCCGAGGCTATCCGGCCGAGGGCAC

GCCTGCCTGGGCGTCACGCCTCGCGTCGCTCCGCGCACCCTGCCCCCCGTCCCGGGGAGG

CGGCGGGCGCAGATGCGGAGATTGGCCCCCCGTGCCTCACGGCGCGGCGGGCCGAAGTGC

GTGCCGCCGGCCGGGACGGACGCGGCGAGTGGTGGACGGACACGTGCGGCGCTCAACGTC

GCCTCCGCCCCCCGGCCCCGGAGGTGCATGCAAGGAACCCACGCCGAGCGCCCCTCGGAA

CACGACCCCAGGTCAG

>ITS_KP058320.1_Liriope_m

CGAACCCGTAAACGCTCCCGCAGGGGCGGAGGGAGGGCGGATATTCCGGCCGCCCGACCC

CGCACCTCGGGGCACAACGGCCGCCCCCGCCCCGCATCGCTGCGGGACGGGCGGCGGGAA

CAACACCCGGCGCGGTGGGCGCCAAGGAACAGTGCTGTCGGAGACCGCCGCGCGCCGGCC

TCGGCGCGTGGCGCGGTCTTTCCATACGTCGAACTTTTACGACTCTCGGCAACGGATATC

TTGGCTCTCGCATCGATGAAGAACGTAGCGAAATGCGATACTTGGTGTGAATTGCAGAAT

CCCGTGAACCATCGAGTCTTTGAACGCAAGTTGCGCCCGAGGCTATCCGGCCGAGGGCAC

GCCTGCCTGGGCGTCACGCCTCGCGTCGCTCCGCGCACCCTGCCCCCCGTCCCGGGGAGG

CGGCGGGCGCAGATGCGGAGATTGGCCCCCCGTGCCTCACGGCGCGGCGGGCCGAAGTGC

GTGCCGCCGGCCGGGACGGACGCGGCGAGTGGTGGACGGACACGTGCGGCGCTCAACGTC

GCCTCCGCCCCCCGGCCCCGGAGGTGCATGCAAGGAACCCACGCCGAGCGCCCCTCGGAA

CACGACCCCAGGTCAG

>ITS_JF327833.1_Liriope_m

CGAACCCGTAAACGCTCCCGCAGGGGCGGAGGGAGGGCGGATATTCCGACCGCCCGACCC

CGCACCTCGGGGCACAACGGCCGCCCCCGCCCCGCATCGCTGCGGGACGGGCGGCGGGAA

CAACACCCGGCGCGGTGGGCGCCAAGGAACAGTGCTGTCGGAGACCGCCGCGCGCCGGCC

TCGGCGCGTGGCGCGGTCTTTCCATACGTCGAACTTTTACGACTCTCGGCAACGGATATC

TTGGCTCTCGCATCGATGAAGAACGTAGCGAAATGCGATACTTGGTGTGAATTGCAGAAT

CCCGTGAACCATCGAGTCTTTGAACGCAAGTTGCGCCCGAGGCTATCCGGCCGAGGGCAC

GCCTGCCTGGGCGTCACGCCTCGCGTCGCTCCGCGCACCCTGCCCCCCGTCCCGGGGAGG

CGGCGGGCGCAGATGCGGAGATTGGCCCCCCGTGCCTCACGGCGCGGCGGGCCGAAGTGC

GTGCCGCCGGCCGGGACGGACGCGGCGAGTGGTGGACGGACACGTGCGGCGCTCAACGTC

GCCTCCGCCCCCCGGCCCCGGAGGTGCATGCAAGGAACCCACGCCGAGCGCCCCTCGGAA

CA

>ITS_KF671308.1_Liriope_m

TTGCATCGATGAAGAACGCAGCGAAATGCGATACTTGGTGTGAATTGCAGAATCCCGTGA

ACCATCGAGTCTTTGAACGCAAGTTGCGCCCGAGGCTATCCGGCCGAGGGCACGCCTGCC

TGGGCGTCACGCCTCGCGTCGCTCCGCGCACCCTGCCCCCCGTCCCGGGGAGGCGGCGGG

CGCAGATGCGGAGATTGGCCCCCCGTGCCTCAAGGCGCGGCGGGTCGAAGTGCGTGCCGC

CGGCCGGGACGGACGCGGCGAGTGGTGGACGGACACGTGCGGCGCTCAACGTCGCATCCG

CCCCCCGGCCCCGGAGGTGCATGCAAGGAACCCACGCCGAGTGCCCCTCGGAACACGACC

CCAGGTCAG

>Lir_654_1324_EM_ITS_ITS1

CGAACCGGCTAACGCTCCCGCAGGGCGGAGGGCGGGCGGATATTCCGACCGCCCGACCCC

GCACCTCGGGGCACAACGGCCGCCCCCGCCCCGCATCGCTCCGGGACGGGCGGCGGGAAC

AACACCCGGCGCGGTGGGCGCCAAGGAACAGTGCTGTCGGAGACCGCCGCGCGCCGGCCT

CGGCGCGTGGCGCGCGTCTTTCCATACGTCGAACTTTTACGACTCTCGGCAACGGATATT

TTGGCTTTTGCATCGATTAAGAACGTAGCGGAATGCGATACTTGGTGTGAATTGCAGAAT

CCCGTGAACCATCGAGTCTTTGAACGCAAGTTGCGCCCGAGGCTATCCGGCCGAGGGCAC

GCCTGCCTGGGCGTCACGCCTCGCGTCGCTTCGCGCACCCTGCCCCCCGTCCCGGGAAGG

CGGCGGGCGCAGATTCGGAGATTGGCCCCCCGTGCCTCACGGCGCGGCGGGCCGAAGTGC

GTGCCGCCAGCCGGGACGGACGCGGCGAGTGGTGGACGGACACGTGCGGCGCTCAACGTC

GCCTCCGCCCCCCGGCCCCGGAGGTGCATGCAAGGAACCCACGCCGAGCGCCCCTGGGAA

CACGACCCCGGGTCAG

>Lir_651_1321_EM_ITS_ITS1

CCCGCAGGGGCGGAGGGAGGGCGGATATTCCGACCGCCCGACCCCGCACCTCGGGGCACA

ACGGCCGCCCCCGCCCCGCATCGCTKCGGGACGGGCGGCGGGAACAACACCCGGCGCGGT

GGGCGCCAAGGAACAGTGCTGTCGGAGACCGCCGCGCGCCGGCCTCGGCGCGTGGCGCGG

TCTTTCCATACGTCGAACTTTTACGACTCTCGGCAACGGATATCTTGGCTCTCGCATCGA

TTAAGAACGTAGCGAAATGCGATACTTGGTGTGAATTGCAGAATCCCGTGAACCATCGAG

TCTTTGAACGCAAGTTGCGCCCGAGGCTATCCGGCCGAGGGCACGCCTGCCTGGGCGTCA

CGCCTCGCGTCGCTYCGCGCACCCTGCCCCCCGTCCCGGGGAGGCGGCGGGCGCAGATGC

GGAGATTGGCCCCCCGTGCCTCACGGCGCGGCGGGCCGAAGTGCGTGCCGCCGGCCGGGA

CGGACGCGGCGAGTGGTGGACGGACACGTGCGGCGCTAACGTCGCCTCCGCCCCCCGGCC

CCGGAGGTGCATGCAAGGA

>Lir_663_1331_EM_ITS_ITS1

CGAACCCGTAACGCTCCCGCAGGGGCGGAGGGAGGGCGGATATTCCGACCGCCCGACCCC

GCACCTCGGGGCACAACGGCCGCCCCCGCCCCGCATCGCTGCGGGACGGGCGGCGGGAAC

AACACCCGGCGCGGTGGGCGCCAAGGAACAGTGCTGTCGGAGACCGCCGCGCGCCGGCCT

CGGCGCGTGGCGCGGTCTTTCTATACATCCAACTTTTACGACTCTCGGCATCGGATATCT

TGGCTCTCGCATCGATGAAGAACGTAGCGAAGTGCGATACTTGGTGTGAATTGCAAAATC

CCGTGAACCATCAAGTCTTTGAACGCGAGTTGCGCCCGAGGCTATCCGGCCGAGGGCACG

CCTGCCTGGGCGTCACGCCTCGCGTCGCTCCGCTCACCCTGCCCCCCGTCCCGGGAAGGC

GGCAGGCGCACATGCCGAGATTGGCCCCCCGTGCCTCACGGCGCGGCGGCCCAAAGTGCG

TGCCACCGGCCGGGACGGACACGGCCAGTGGTGGACGGACACGTGCGGTGCTCAACGTCG

CCTCCACCCCCAGGCCCCGGAGGTGCATGCAAGGAACCCACGCCGAGCGCCCCTCGGAAC

GCGACCCCAGGTCAT

>Lir_647_1276_EM_ITS_ITS1-1

GCGACCCGTAACGCTCCCGCAGGGGCGGAGGGAGGGCGGATATTCCGACCGCCCGACCCC

GCACCTCGGGGCACAACGGCCGCCCCCGCCCCGCATCGCTGCGGGACGGGCGGCGGGAAC

AACACCCGGCGCGGTGGGCGCCAAGGAACAGTGCTGTCGGAGACCGCCGCGCGCCGGCCT

CGGCGCGTGGCGCGGTCTTTCCATACGTCGAACTTTTACGACTCTCGGCAACGGATATCT

TGGCTCTCGCATCGATTAAAAACGTAGCGAAATGCGATACTTTGGTGTGAAATGCAGAAA

CCCGGGGACCAACCAGGCCTTTAACGCAAGTTGCGCCCGAAGCCTTTCCGCCGAAGGCAC

GCCCGCCCGGGCGGCACGCCCTGCGGCGCTTCGCGCAACCCGCCCCCCGGCCCGGGGAGG

CGGCGGGCGCAAAATCGGAGAATGGCCCCCCGTGCCTTAAGGCGCGGCGGGCCGAAGTGC

GTGCCGCCGCCGGGACGGACGCGGCGAGTGGTGGACGGACACGTGCGGCGCTTAACGTCG

CCTCCGCCCCCCGGCCCCGGAGGTGCATGCCAGGAACCCACGCCAAGCGCCCCTTGGAAC

ACGACCCCGGGTCAG

>Lir_664_1332_EM_ITS5p_ITS5p

GGAGGGAGGGCGGATATTCCGACCGCCCGACCCCGCACCTCGGGGCACAACGGCCGCCCC

CGCCCCGCATCGCTGCGGGACGGGCGGCGGGAACAACACCCGGCGCGGTGGGCGCCAAGG

AACAGTGCTGTCGGAGACCGCCGCGCGCCGGCCTCGGCGCGTGGCGCGGTCTTTCCATAC

GTCGAACTTTTACGACTCTCGGCAACGGATATCTTGGCTCTCGCATCGATGAAGAACGTA

GCGAAATGCGATACTTGGTGTGAATTGCAGAATCCCGTGAACCATCGAGTCTTTGAACGC

AAGTTGCGCCCGAGGCTATCCGGCCGAGGGCACGCCTGCCTGGGCGTCACGCCTCGCGTC

GCTCCGCGCACCCTGCCCCCCGTCCCGGGGAGGCGGCGGGCGCAGATGCGGAGATTGGCC

CCCCGTGCCTCACGGCGCGGCGGGCCGAAGTGCGTGCCGCCGGCCGGGACGGACGCGGCG

AGTGGTGGACGGACACGTGC

>Lir_670_1338_EM_ITS5p_ITS5p

ATTCCGACCGCCCGACCCCGCACCTCGGGGCACAACGGCCGCCCCCGCCCCGCATCGCTG

CGGGACGGGCGGCGGGAACAACACCCGGCGCGGTGGGCGCCAAGGAACAGTGCTGTCGGA

GACCGCCGCGCGCCGGCCTCGGCGCGTGGCGCGGTCTTTCCATACGTCGAACTTTTACGA

CTCTCGGCAACGGATATCTTGGCTCTCGCATCGATGAAGAACGTAGCGAAATGCGATACT

TGGTGTGAATTGCAGAATCCCGTGAACCATCGAGTCTTTGAACGCAAGTTGCGCCCGAGG

CTATCCGGCCGAGGGCACGCCTGCCTGGGCGTCACGCCTCGCGTCGCTCCGCGCACCCTG

CCCCCCGTCCCGGGGAGGCGGCGGGCGCAGATGCGGAGATTGGCCCCCCGTGCCTCACGG

CGCGGCGGGCCGAAGTGCGTGCCGCCGGCCGGGACGGACGCGGCGAGTGGTGGACGGACA

>Lir_668_1336_EM_ITS5p_ITS5p

GCGGAGGGAGGGCGGATATTCCGACCGCCCGACCCCGCACCTCGGGGCACAACGGCCGCC

CCCGCCCCGCATCGCTGCGGGACGGGCGGCGGGAACAACACCCGGCGCGGTGGGCGCCAA

GGAACAGTGCTGTCGGAGACCGCCGCGCGCCGGCCTCGGCGCGTGGCGCGGTCTTTCCAT

ACGTCGAACTTTTACGACTCTCGGCAACGGATATCTTGGCTCTCGCATCGATGAAGAACG

TAGCGAAATGCGATACTTGGTGTGAATTGCAGAATCCCGTGAACCATCGAGTCTTTGAAC

GCAAGTTGCGCCCGAGGCTATCCGGCCGAGGGCACGCCTGCCTGGGCGTCACGCCTCGCG

TCGCTCCGCGCACCCTGCCCCCCGTCCCGGGGAGGCGGCGGGCGCAGATGCGGAGATTGG

CCCCCCGTGCCTCACGGCGCGGCGGGCCGAAGTGCGTGCCGCCGGCCGGGACGGACGCGG

CGAGTGGTGGACGGACACGT

>Lir_631_1261_EM_ITS_ITS1

CAACGGCCGCCCCCGCCCCGCATCGCTGCGGGACGGGCGGCGGGAACAACACCCGGCGCG

GTGGGCGCCAAGGAACAGTGCTGTCGGAGACCGCCGCGCGCCGGCCTCGGCGCGTGGCGC

GGTCTTTCCATACGTCGAACTTTTACGACTCTCGGCAACGGATATCTTGGCTCTCGCATC

GATGAAGAACGTAGCGAAATGCGATACTTGGTGTGAATTGCARAATCCCGTGAACCATCK

AGTCTTTGAACGCAAGTTGCGCCCGAGGCTATCCGGCCGAGGGCACGCCTGCCTGGGCGT

CACGCCTCGCGTCGCTCCGCGMACCCTGCCCCCCGTCCCGGGGAGGCGGCGGGCGCACAT

KCGGAGATTGGCCCCCCGTGCCTCAYGGCGCGCCGGGACAAATTGCGTGCCGCCGGYCGG

GACGGACGCGGCGAGTGGTGGACGGACACGTGCGGCGCTCAACGTCGCCTCCGCCCCCCG

GCCCCGGAGGTGCATGCAAGGAACCCACGCCGAGCGCCCCTCGGAACACGACCCCAGGT

>Lir_669_1337_EM_ITS5p_ITS5p

CGGAGGGAGGGCGGATATTCCGACCGCCCGACCCCGCACCTCGGGGCACAACGGCCGCCC

CCGCCCCGCATCGCTGCGGGACGGGCGGCGGGAACAACACCCGGCGCGGTGGGCGCCAAG

GAACAGTGCTGTCGGAGACCGCCGCGCGCCGGCCTCGGCGCGTGGCGCGGTCTTTCCATA

CGTCGAACTTTTACGACTCTCGGCAACGGATATCTTGGCTCTCGCATCGATGAAGAACGT

AGCGAAATGCGATACTTGGTGTGAATTGCAGAATCCCGTGAACCATCGAGTCTTTGAACG

CAAGTTGCGCCCGAGGCTATCCGGCCGAGGGCACGCCTGCCTGGGCGTCACGCCTCGCGT

CGCTCCGCGCACCCTGCCCCCCGTCCCGGGGAGGCGGCGGGCGCAGATGCGGAGATTGGC

CCCCCGTGCCTCACGGCGCGGCGGGCCGAAGTGCGTGCCGCCGGCCGGGACGGACGCGGC

AAGTGGTGGACGGACACGTG

>Lir_676_1341_EM_ITS5p_ITS5p

CGGAGGGAGGGCGGATATTCCGACCGCCCGACCCCGCACCTCGGGGCACAACGGCCGCCC

CCGCCCCGCATCGCTGCGGGACGGGCGGCGGGAACAACACCCGGCGCGGTGGGCGCCAAG

GAACAGTGCTGTCGGAGACCGCCGCGCGCCGGCCTCGGCGCGTGGCGCGGTCTTTCCATA

CGTCGAACTTTTACGACTCTCGGCAACGGATATCTTGGCTCTCGCATCGATGAAGAACGT

AGCGAAATGCGATACTTGGTGTGAATTGCAGAATCCCGTGAACCATCGAGTCTTTGAACG

CAAGTTGCGCCCGAGGCTATCCGGCCGAGGGCACGCCTGCCTGGGCGTCACGCCTCGCGT

CGCTCCGCGCACCCTGCCCCCCGTCCCGGGGAGGCGGCGGGCGCAGATGCGGAGATTGGC

CCCCCGTGCCTCACGGCGCGGCGGGCCGAAGTGCGTGCCGCCGGCCGGGACGGACGCGGC

GAGTGGTGGACGGACACGTG

>Lir_675_1340_EM_ITS5p_ITS5p

ATATTCCGACCGCCCGACCCCGCACCTCGGGGCACAACGGCCGCCCCCGCCCCGCATCGC

TGCGGGACGGGCGGCGGGAACAACACCCGGCGCGGTGGGCGCCAAGGAACAGTGCTGTCG

GAGACCGCCGCGCGCCGGCCTCGGCGCGTGGCGCGGTCTTTCCATACGTCGAACTTTTAC

GACTCTCGGCAACGGATATCTTGGCTCTCGCATCGATGAAGAACGTAGCGAAATGCGATA

CTTGGTGTGAATTGCAGAATCCCGTGAACCATCGAGTCTTTGAACGCAAGTTGCGCCCGA

GGCTATCCGGCCGAGGGCACGCCTGCCTGGGCGTCACGCCTCGCGTCGCTCCGCGCACCC

TGCCCCCCGTCCCGGGGAGGCGGCGGGCGCAGATGCGGAGATTGGCCCCCCGTGCCTCAC

GGCGCGGCGGGCCGAAGTGCGTGCCGCCGGCCGGGACGGACGCGGCGAGTGGTGGACGGA

C

>Lir_641_1270_SP_ITS1_ITS1

GACCCCGCAGCTCGGGGCACAACGGCCGCCCCCGCCCCGCATCGCTKCGGGACGGGCGGC

GGGAACAACACCCGGCGCGGTGGGCGCCAAGGAACAGTGCTGTCGGAGACCGCCGCGCGC

CGGCCTCGGCGCGTGGCGCGGTCTTTCCATACGTCGAACTTTTACGACTCTCGGCHACGG

ATATCTTGGCTCTCGCATCGATKAAKAACGTAGCGAARTGCGATACTTGGTGTGAATTGC

AGAATCCCGTGAACCRTCGAGTCTTTGAACGCAMGTTGCGCCCGAGGCTATCCGGCCGAG

GGCACGCCTGCCTGGGCGTCACGCCTCGCGTCGCTCCGCGCASGCTGCCCCCCGTCCCGG

GGAGGCGGCGGGCGCAGATGCGGAGATTGRCCCCCMGTGCCTCAGGGYTCGGCGGGCCGA

AGTGCGTGCCGCCGGCCGGGACGRACGCGGCGAGTGGTGGACGGACACGTGCGGCGCTCA

ACGTCGCCTCCGCCCCCCGGCCCCGGAGGTGCATGCAAG

>Lir_656_1326_EM_ITS5p_ITS5p

CGGCCGCCCGACCCCGCACCTCGGGGCACAACGGCCGCCCCCGCCCCGCATCGCTGCGGG

ACGGGCGGCGGGAACAACACCCGGCGCGGTGGGCGCCAAGGAACAGTGCTGTCGGAGACC

GCCGCGCGCCGGCCTCGGCGCGTGGCGCGGTCTTTCCATACGTCGAACTTTTACGACTCT

CGGCAACGGATATCTTGGCTCTCGCATCGATGAAGAACGTAGCGAAATGCGATACTTGGT

GTGAATTGCAGAATCCCGTGAACCATCGAGTCTTTGAACGCAAGTTGCGCCCGAGGCTAT

CCGGCCGAGGGCACGCCTGCCTGGGCGTCACGCCTCGCGTCGCTCCGCGCACCCTGCCCC

CCGTCCCGGGGAGGCGGCGGGCGCAGATGCGGAGATTGGCCCCCCGTGCCTCACGGCGCG

GCGGGCCGAATTGCGTGCCGCCCGCCGGGACG

>Lir_649_1320_EM_ITS_ITS1

TGCGATACTTGGTGTGAATTGCAGAATCCCGTGAACCATCGAGTCTTTGAACGCATGTTG

CGCCCGAGGCTATCCGGCCGAGGGCACGCCTGCCTGGGCGTCACGCCTCGCGTCGCTTCG

CGCACCCTGCCCCCCGTCCCGGGGAGGCGGCGGGCGCAGATCGGAGATTGGCCCCCCGTG

CCTCACGGYGCGRCGGGCCGAAGTGCGTGCCGCCTGCCGGGACGGAYGCGGCDAGTGKTG

GACGGACACGTGCGGCGCTTAACGTCGCCTCCGCCCCCCGGCCCCCGAWGTGCATGCAAG

GAATCCACGCCAAGCGCCCCTTKAAACACGACCCCGGGTCAT

>Lir_640_1269_SP_ITS1_ITS1

GGCGGATATTCCGACCGCCCGACCCCGCACCTCGGGGCACAACGGCCGCCCCCGCCCCGC

ATCGCTGCGGGACGGGCGGCGGGAACAACACCCGGCGCGGTGGGCGCCAAGGAACAGTGC

TGTCGGAGACCGCCGCGCGCCGGCCTCGGTKCGTGKCGCGGTCTTTCYWTACATCSAACT

TTTACGACTCTYGGCCWCGGAYATCTTGGCTCTCGCWTCGATRAAGAACGTATCGAAGTG

CGATACTTGGTGTGAATTGCARAATCCCCTGWACCATCGAGTCTTTGAACGCGCGTTGCG

CCCGAGGCTATSCSGGCGAGGGCACGCCTGCCTGGGCGTCWCGCCTCGCGTCGCTCCGCG

YACCCTGCMCSCCGTCCCGGGGARGCCGCRGRCGCACATGCGGAGATTGACCCCCCGTGC

CTCACRGCTCGGCGGCGCCAACTGCGTGCCACCCGCCGAGACGGACACGGCSAGTGGTGG

ACCKACACGTGCGGYCCTCAACGTCGCCTCCGCCCCCCG

>Liri_673_EM_ITS_5P_ITS_5P

CGCCCGACCCCGCACCTCGGGGCACAATGGCCGCCCCCGCCCCGCATCGCTGCGGGACGG

GCGGCGGGAACAACACCCGGCGCGGTGGGCGCCAAGGAACAGTGCTGTCGGAGACCGCCG

CGCGCCGGCCTCGGCTCGTGGCGCGGTCTTTCCATACATCGAACTTTTACGACTCTCGGC

AACGCATATCTTGGCTCTCGCATCGATGATGAACTTATCGAAATGCGATACTTGGTGTGA

ATTGCAGAATCCCTTTAACCATCGAGTCTTTGAACACGCTTTGCGCCCGAGGCTATCCCG

GCCAGGGCACCCCTGCCTGGGCGTCTCGCCTCGCGTCGCTCCGCGTACCCTGCCCCCCGT

CCCGGGGAATCCGCAGATTCACATCCGGAGATTGACCCCCCGTGCCTCACAACTCCGTGC

CTCCAACTGCGACCCACCCGCCGAGACGTACACGGACAC

>Lir_645_1274_EM_ITS_ITS1

TCGGGGCACAACGGCCGCCCCCGCCCCGCATCGCTGCGGGACGGGCGGCGGGAACAACAC

CCGGCGCGGTGGGCGCCAAGGAACAGTGCTGTCGGAGACCGCCGCGCGCCGGCCTCGGCG

CGTGGCGCGGTCTTTCCATACGTCGAACTTTTACGACTCTCGGCAACGGATTTTTGGCTC

TCGCATCGATTAAGAACGTAGCGAAATGCGATACTGGGGGTGAATTGCAGAATCCCGGGG

ACCATTGAGTCTTTTAACGCAAGTTGCGCCCGAGGCTTTTCGGCCGAGGGCACGCCCGCC

TGGGCGTCACGCCTCAGGTCGGTTCGGGCACCCTGGCCCCCGACCCGGGGAAGCGGCCGG

CGCAGAAGCGGAGAATGGCCCCCCGGGCCTTACGGCGCGGCGGGCCGAAATGCGTGCCGC

C

>Lir_638_1267_EM_ITS_ITS1

AACACCCGGCGCGGTGGGCGCCAAGGAACAGTGCTGTCGGAGACCGCCGCGCGCCGGCCT

CGGCGCGTGGCGCGGTCTTTCCATACGTCGAACTTTTACAACTCTCGGCAACGGATATCT

TGGCTCTCGCATCGATGAAGAACGTAGCGAAATGCGATACTTGGTGTGAATTGCAGAATC

CCGTGAACCATCGAGTCTTTGAACGCAAGTTGCGCCCGAGGCTATCCGGCCGAGGGCACG

CCTGCCTGGGCGTCACGCCTCGCGTCGCTCCGCGCACCCTGCCCCCCGTCCCGGGGAGG

>Lir_674_1339_EM_ITS5p_ITS5p

CTTCCCGACCCCGCACCTCGGGCCACAACGGCCGCCCCCGCCCCGCATCGCTGCGGGACG

GGCGGCGGGAACAACACCCGGCGCGGTGGGCGCCAAGGAACAGTGCTGTCGGAGACCGCC

GCGCGCCGGCCTCTGCGCGTGGCGTGTCTTTCCTTACGTCGAACTTTTACGACTCTCGGC

AACGGATATTTTGGCTCTCGCATCTATTAAGAACGTAGCGAAATGCGATACTTGGTGTGA

ATTGCAGAATCCCGTGAACCATCGAGTCTTTGAACGCAAGTTTCCCTCTAAGCTATC

>Lir_642_1271_SP_ITS1_ITS1

GAGGGCGGATATTCCGACCGCCCGACCCCGCACCTCGGGGCACAACGGCCGCCCCCGCCC

CGCATCGCTGCGGGACGGGCGGCGGGAACAACACCCGGCGCGGTGGGCGCCAAGGAACAG

TGCTGTCGGAGACCGCCGCGCGCCGGCCTCGGCGCGTGGCGCGGTCCTTCCATACGTCGA

ACTTTTACGACTCTCGGCAACGGATATCTTGGCTCTCGCATCGATGAAGAACGTAGCGAA

ATGCGATACTTGGTGTGAATTGCAGAATCCCGTGAACCATCGAGTCTTTGAACGCAAGTT

GCGCCCGAGGCTATCCGGCCGAGGGCACGCCTGCCTGGGCGTCACGCCTCGCGTCGCTCC

GCGCACCCTGCCCCCCGTCCCGGGGAGGCGGCGGGCGCAGATGCGGAGATTGGCCCCCCG

TGCCTCACGGCGCGGCGGGCCGAAGTGCGTGCCGCCGGCCGGGACGGACGCGGCGAGTGG

TGGACGGACACGTGCGGCGCTCAACGTCGCCTCCGCCCCCCGGCCCCGGAGGTGCATGCA

>Lir_637_1266_SP_ITS1_ITS1

GCGGATATTCCGACCGCCCGACCCCGCACCTCGGGGCACAACGGCCGCCCCCGCCCCGCA

TCGCTGCGGGACGGGCGGCGGGAACAACACCCGGCGCGGTGGGCGCCAAGGAACAGTGCT

GTCGGAGACCGCCGCGCGCCGGCCTCGGCGCGTGGCGCGGTCCTTCCATACGTCGAACTT

TTACGACTCTCGGCAACGGATATCTTGGCTCTCGCATCGATGAAGAACGTAGCGAAATGC

GATACTTGGTGTGAATTGCAGAATCCCGTGAACCATCGAGTCTTTGAACGCAAGTTGCGC

CCGAGGCTATCCGGCCGAGGGCACGCCTGCCTGGGCGTCACGCCTCGCGTCGCTCCGCGC

ACCCTGCCCCCCGTCCCGGGGAGGCGGCGGGCGCAGATGCGGAGATTGGCCCCCCGTGCC

TCACGGCGCGGCGGGCCGAAGTGCGTGCCGCCGGCCGGGACGGACGCGGCGAGTGGTGGA

CGGACACGTGCGGCGCTCAACGTCGCCTCCGCCCCCCGGCCCCGGAGGTGCATGCAAGGA

>Lir_639_1268_EM_ITS_ITS1

ACGGCCGCCCCCGCCCCGCATCGCTGCGGGACGGGCGGCGGGAACAACACCCGGCGCGGT

GGGCGCCAAGGAACAGTGCTGTCGGAGACCGCCGCGCGCCGGCCTCGGCGCGTGGCGCGG

TCCTTCCATACGTCGAACTTTTACGACTCTCGGCAACGGATATCTTGGCTCTCGCATCGA

TGAAGAACGTAGCGAAATGCGATACTTGGTGTGAATTGCAGAATCCCGTGAACCATCGAG

TCTTTGAACGCAAGTTGCGCCCGAGGCTATCCGGCCGAGGGCACGCCTGCCTGGGCGTCA

CGCCTCGCGTCGCTCCGCGCACCCTGCCCCCCGTCCCGGGGAGGCGGCGGGCGCAGATGC

GGAGATTGGCCCCCCGTGCCTCACGGCGCGGCGGGCCGAAGAGCGTGCCGCCGGCCGGGA

CGGACGCGGCGAGTGGTGGACGGACACGTGCGGCGCTCAACGTCGCCTCCGCCCCCCGGC

CCCGGAGGTGCATGCAAGGAACCCACGCCGAGCGCCCCTCGGAACACGACCCCAGGTCAG

>Lir_653_1323_EM_ITS_ITS1

CGGAACCCGTAACGCTCCCGCAGGGGCGGAGGGAGGGCGGATATTCCGACCGCCCGACCC

CGCACCTCGGGGCACAACGGCCGCCCCCGCCCCGCATCGCTGCGGGACGGGCGGCGGGAA

CAACACCCGGCGCGGTGGGCGCCAAGGAACAGTGCTGTCGGAGACCGCCGCGCGCCGGCC

TCGGCGCGTGGCGCGGTCCTTCCATACGTCGAACTTTTACGACTCTCGGCAACGGATATC

TGGCTCTTGCATCGATTAAGAAGTAGCGAAATGCGATACTTGGTGTGAATTGCAGAAACC

CGGGAACCATCGAGTCTTTGAACGCAAGTTGCGCCCGAGGCTTTTCGGCCGAGGGCACGC

CCGCCTGGGCGTCACGCCCCGCGGCGCTTCGCGCACCCCGCCCCCCGTCCCGGGGAGGCG

GCCGGCGCAGATTCGGAGATTGGCCCCCCGTGCCTCAGGGCGCGGCGGGCCGAAGTGCGT

GCCGCCGCCGGGACGGACGCGGCGAGTGGTGGACGGACACGTGCGGCGCTTAACGTCGCC

CCCGCCCCCCGGCCCCGGAGGTGCATGCAAGGAACCCACGCCGAGCGCCCCTTGGAACAC

GACCCCAGGTCAG

>Lir_662_1330_EM_ITS_ITS1

ACCCTGTATACGCCTCTCTGCATGGGCGGATGGTGAGTGGCGTGAATCCGACCGCCCGAC

CCCGCACCGGTGGCACAACGGCCGCCCCCGCCCCGCATCGCGCGGGACGGGCGGCGGGAA

CAACACCCGGCGCGGTGGGCGCCAAGGAACAGTGCTGTCGGAGACCGCCGCGCGCCGGCC

TCGGCGCGTGGCGCGGTCCTTCCATACGTCGAACTTTTACGACTCTCGGCAACGGATATC

TGGCTCTCGCATCGATTAAGAACGTAGCGAAATGCGATACTTGGTGTGAATTGCAGAATC

CCGTGAACCATCGAGTCTTTGAACGCAAGTTGCGCCCGAGGCTATCCGGCCGAGGGCACG

CCTGCCTGGGCGTCACGCCTCGCGTCGCTTCGCGCACCCTGCCCCCCGTCCCGGGGAGGC

GGCGGGCGCAGATGCGGAGATTGGCCCCCCGTGCCTCAGGGCGCGGCGGGCCGAATAGCG

TGCCGCCGCCGGGACGGACGCGGCGAGTGGTGGACGGACACGTGCGGCGCTTAACGTCGC

CTCCGCCCCCCGGCCCCGGAGGTGCATGCAAGGAACCCACGCCAAGCGCCCCTCGGAACA

CGACCCCAGGTCAG

>Lir_655_1325_EM_ITS_ITS1

ACGGCCGCCCCCGCCCCGCATCGCTGCGGGACGGGCGGCGGGAACAACACCCGGCGCGGT

GGGCGCCAAGGAACAGTGCTGTCGGAGACCGCCGCGCGCCGGCCTCGGCGCGTGGCGCGG

TCCTTCCATACGTCGAACTTTTACGACTCTCGGCAACGGATATCTTGGCTCTCGCATCGA

TGAAGAACGTAGCGAAATGCGATACTTGGTGTGAATTGCAGAATCCCGTGAACCATCGAG

TCTTTGAACGCAAGTTGCGCCCGAGGCTATCCGGCCGAGGGCACGCCTGCCTGGGCGTCA

CGCCTCGCGTCGCTCCGCGCACCCTGCCCCCCGTCCCGGGGAGGCGGCGGGCGCAGATGC

GGAGATTGGCCCCCCGTGCCTCACGGCGCGGCGGGCCGAAGTGCGTGCCGCCGGCCGGGA

CGGACGCGGCGAGTGGTGGACGGACACGTGCGGCGCTCAACGTCGCCTCCGCCCCCCGGC

CCCGGAGGTGCATGCAAGGA

>Lir_660_1328_EM_ITS_ITS1

CGAACCGTAACGCTCCCGCAGGGGCGGAGGGAGGGCGGATATTCCGACCGCCCGACCCCG

CAACTCGGGGCACAACGGCCGCCCCCGCCCCGCATCGCTCGGGACGGGCGGCGGGAACAA

CACCCGGCGCGGTGGGCGCCAAGGAACAGTGCTGTCGGAGACCGCCGCGCGCCGGCCTCG

GCGCGTGGCGCGGTCCTTCCATACGTCGAACTTTTACGACTCTCGGCAACGGGATATCTT

GGCTTTCGCAACGATTAAAAACGTAGCGAAATGCGATTCTTGGTGTGAATTGCAGAATCC

CGTGAACCATCGAGTCTTTGAACGCAAGTTGCGCCCGAGGCTATCCGGCCGAGGGCACGC

CTGCCTGGGCGTCACGCCTCGCGACGCTTCGCGAAGCCGGCCCCCCGTCCCGGGGAGGCG

GCGGGCGCAGATTCGGAGATTGGCCCCCCGGGCCTCAAGGCGCGGCGGGCCGAAGAGCGT

GCCGCCGGCCGGGACGGACACGGCAAGGGGTGGACGGACACGTGCGGCGCTCAACGTCGC

CACCGCCCCCCGGCCCCGGAGGTGCATGCAAGGAACCCACGCCGAGCGCCCCTCGAAACA

CGACCCCGGGTCAA

>Lir_634_1264_EM_ITS_ITS1

GGGCGGATATTCCGACCGCCCGACCCCGCACCTCGGGGCACAACGGCCGCCCCCGCCCCG

CATCGCTGCGGGACGGGCGGCGGGAACAACACCCGGCGCGGTGGGCGCCAAGGAACAGTG

CTGTCGGAGACCGCCGCGCGCCGGCCTCGGCGCGTGGCGCGGTCCTTCCATACGTCGAAC

TTTTACGACTCTCGGCAACGGATATCTTGGCTCTCGCATCGATGAAGAACGTAGCGAAAT

GCGATACTTGGTGTGAATTGCAGAATCCCGTGAACCATCGAGTCTTTGAACGCAAGTTGC

GCCCGAGGCTATCCGGCCGAGGGCACGCCTGCCTGGGCGTCACGCCTCGCGTCGCTCCGC

GCACCCTGCCCCCCGTCCCGGGGAGGCGGCGGGCGCAGATGCGGAGATTGGCCCCCCGTG

CCTCACGGCGCGGCGGGCCGAAGTGCGTGCCGCCGGCCGGGACGGACGCGGCTAGTGGTG

GACGGACACGTGCGGCGCTCAACGTCACCTCCGCCCCCCG

>Lir_712_1384_EM_ITS5p_ITS5p

CGACCGCCCGACCCCGCACCTCGGGGCACAACGGCCGCCCCCGCCCCGCATCGCTGCGGG

ACGGGCGGCGGGAACAACACCCGGCGCGGTGGGCGCCAAGGAACAGTGCTGTCGGAGACC

GCCGCGCGCCGGCCTCGGCGCGTGGCGCGGTCCTTCCATACGTCGAACTTTTACGACTCT

CGGCAACGGATATCTTGGCTCTCGCATCGATGAAGAACGTAGCGAAATGCGATACTTGGT

GTGAATTGCAGAATCCCGTGAACCATCGAGTCTTTGAACGCAAGTTGCGCCCGAGGCTAT

CCGGCCGAGGGCACGCCTGCCTGGGCGTCACGCCTCGCGTCGCTCCGCGCACCCTGCCCC

CCGTCCCGGGGAGGCGGCGGGCGCAGATGCGGAGATTGGCCCCCCGTGCCTCACGGCGCG

GCGGGCCGAAGTGCGTGCCGCCGGCCGGGACGGACGCGGCGAGTGGTGGACGGACACGTG

C

>Lir_643_1272_EM_ITS5pM_ITS5p

AACGGCCGCCCCCGCCCCGCATCGCTGCGGGACGGGCGGCGGGAACAACACCCGGCGCGG

TGGGCGCCAAGGAACAGTGCTGTCGGAGACCGCCCCGCGCCGGCCTCGGCGCGTGGCGCG

GTCCTTCCTTACATCGAAGTTTTACGACTTTTGGCCACGGACATCTTGGCTCTCGCATCG

ATAAAGAACGTATCGAAGTGCGATACTTGGTGTGAATTGCAGAATCCCGTGTACCATCGA

GTCTTTGAACGCAAGTTGCGCCCGAGGCTATSCCGGCGAGGGCACGCCTGCCTGGGCGTC

ACGCCTCGCGTCGCTCCGCGCACCCTGCCCCCCGTCCCGGGGAAGCCGCAGGCGCAGATG

CGGAGATTGACCCCCCGTGCCTCAGAGCGCGGCGGGCCCAGCAGGGTGCCGCCCGCCGAG

AGGGACACGGACACTGGTG

>Ophio_711_1379_EM_ITS_ITS1

CCCGCATCGCTTCGGGACGGGCGGCGGGAACAACACCCGGCGCGCTGGGCGCCCAGGAAC

CGTGCTGTCGGAGACCGCCGCGCGCCGGCCTCGGCGCGTGGCGCGGTCCTTCCATCGTCG

GAATTTTCGAACTCTGGGCACGGAATATTTTGGCTTCTGCATCGGATAAGAACGGAGCGG

AATTCGAAACTTTGGGTTAATTTCAGAAACCCGAGGAACGATGCGGCTTTTAACCCAAGG

TGCGCCCGAAGCCTATCGGCCGAGGCACACCTGCCTGGGCGGAACGCCTGGCGCCGGTTC

GCGCAACCAGCCCCCCGACCCGGGGAAGGCGCGGGCACCGATTCGGAGATTGGCCCCCCG

TGGCTCACGGAGCGGCGGGCCCAAATGCGTGTCGCCCGACGAGAACGACGCGGCGAGTG

>Ophio_693_1365_EM_ITS_ITS1

CTCCCGCAGGGGCGGAGGGAGGGCGGATATTCCGACCGCCCGACCCCGCACCTCGGGGCA

CAACGGCCGCCCCCGCCCCGCATCGCTTCGGGACGGGCGGCGGGAACAACACCCGGCGCG

GTGGGCGCCAAGGAACAGTGCTGTCGGAGGCCGCCGCGCGCCGGCCTCGGCGCGTGGAGC

GGTCTTTCCATACGTCGAACTTTTACGACTCTCGGCAACGGATATTCTTGGCTCTGCATC

GATTAAGAACGGAGCGGAATGCGATACTTGGTGTGAATTGCAGAAACCCGTGAACCATCG

AGTCTTTGAACGCAAGTTGCGCCCGAGGCTATCCGGCCGAGGGCACGCCTGCCTGGGCGT

CACGCCCCGCGTCGCTTCGCGCACCCCGCCCCCCGGCCCGGGGAGGCGGCGGGCGCAGAT

TCGGAGATTGGCCCCCCGGGCCTCACGGCGCGGCGGGTCGAAGTGCGTGCCGCCTGCCGG

GACGGACGCGGCGAGTGGTGGACGGACACGTGCGGCGCTTAACGTCGCCTCCGCCCCCCG

GCCCCGGAAGTGCATGCA

>Ophio_695_1366_EM_ITS_ITS1

CGCCCCCGCCCCGCATCGCTGCGGGACGGGCGGCGGGAACAACACCCGGCGCGGTGGGCG

CCAAGGAACAGTGCTGTCGGAGGCCGCCGCGCGCCGGCCTCGGCGCGTGGAGCGGTCTTT

CCATACGTCGAACTTTTACGACTCTCGGCAACGGATATCTTGGCTCTCGCATCGATTAAG

AACGTAGCGAAATGCGATACTTGGTGTGAATTGCAGAATCCCGTGAACCATCGAGTCTTT

GAACGCAAGTTGCGCCCGAGGCTATCCGGCCGAGGGCACGCCTGCCTGGGCGTCACGCCT

CGCGTCGCTTCGCGCACCCTGCCCCCCGTCCCGGGGAGGCGGCGGGCGCAGATCCGGAGA

TTGGCCCCCCGTGCCTCACGGCGCGGCGGGTCGAAGTGCGTGCCGCCTGCCGGGACGGAC

GCGGCGAGTGGTGGACGGACACGTGCGGCGCTTAACGTCGCCTCCGCCCCCCGGCCCCGG

AGGTGCATGCAAGGAACCCA

>Ophio_709_1378_EM_ITS5p_ITS5p

AAACGCTCCCGCAGGGGCGGAGGGAGGGCGGATATTCCAACCGCCCGACCCCGCACCTCG

GGGCTCAATGGCCGCCCCGCCCCGCAACGCTTCCAGACCGGCGGCGGGAACAACACCCAG

CGCGGTGGGCGCCAAGGAACATGGCTTCMGAAAGCGCGCCGGCGTCGGCGCGCAGCGCGA

TGTCATTTCCTTTCTTCGAACTTTTACTATTCTCAGCAACGGATATCTTGGCTCTGGTAT

CGATGAAGAACATAGCGGAATGCGATACTTTGTGTGAAGGTGAGAATCCCGTGAACCATC

GCGTCTTTGAACGCAAGTTGCGCCTGAGGCTATCCGGCCGAGGTCAWGCCTGCCTGGGCG

TCATGCCTCAGCTCATTGCGTGCACCTTGCCCCCKCCTGGGGAGGCGGCGAGCACACATG

CAGAGATTGGCCCCCCGTGCC

>ITS_KF671305.1_Liriope_g

CGAACCCGTAAACGCTCCTGCAGGGGCGGAGGGAGGGCGGATATTCCGACCGCCCGACCC

CGCACCTCGGGGCTCAATGGCCGCCCCCGCCCCGCATCGCTGCGGGACGGGCGGCGGGAA

CAACACCCGGCGCGGTGGGCGCCAAGGAACAGTGCTGTCGGAGAGCGCGCCGCGCGCCGG

CCTCGGCGCGCAGCGCGGTCTTTCCATACGTCGAACTTTTACTACTCTCGGCAACGGATA

TCTTGGCTCTCGCATCGATGAAGAACGCAGCGAAATGCGATACTTGGTGTGAATTGCAGA

ATCCCGTGAACCATCGAGTCTTTGAACGCAAGTTGCGCCCGAGGCTATCCGGCCGAGGGC

ACGCCTGCCTGGGCGTCACGCCTCGCGTCGCTCCGCGCACCCTGCCCCCCGTCATGGGGA

GGCGGCGGGCGCAGATGCGGAGATTGGCCCCCCGTGCCTGACGGCGCGGCGGGTCGAAGT

GCGTGCCGCCGGCCGGGACGGACGCGGCGAGTGGTGGACGGACACGTGCGGCGCTCAACG

TCGCCTCCGCCCCCCGGCCCCGGAGGTGCATGCAAGGAACCCACGCCGAGCACCCCTCGG

AACACGACCCCAGGTCAG

>ITS_KF671304.1_Liriope_g

CGAACCCGTAAACGCCCCTGCAGGGGCGGAGGGAGGGCGGATATTCCGACCGCCCGACCC

CGCACCTCGGGGCTCAATGGCCGCCCCCGCCCCGCATCGCTGCGGGACGGGCGGCGGGAA

CAACACCCGGCGCGGTGGGCGCCAAGGAACAGTGCTGTCGGAGAGCGCGCCGCGCGCCGG

TCTCGGCGCGCAGCGCGATCTTTCCATACGTCGAACTTTACGACTCTCGCAACGGATTGC

ATCGATGAAGAACGCAGCGAAATGCGATACTTGGTGTGAATTGCAGAATCCCGTGAACCA

TCGAGTCTTTGAACGCAAGTTGCGCCCGAGGCTATCCGGCCGAGGGCACGCCTGCCTGGG

CGTCACGCCTCGCGTCGCTCCGCGCACCCTGCCCCCCGTCCTGGGGAGGCGGCGGGCGCA

GATGCGGAGATTGGCCCCCCGTGCCTGACGGCGCGGCGGGTCGAAGTGCGTGCCGCCGGC

CGGGAAGGACGCGACGAGTGGTGGACGGACACGTGCGGCGCTCAACGTCGCCTCCGCCCC

CCGGCCCCGGAGGTGCATGCAAGGAACCCTGG

>ITS_KF671244.1_Ophiopogon_c

CGAACCCGTAAACGCTCCTGCAGGGGCGGAGGGAGGGCGGATATTCAGACCGTCCGACCA

CGCACCTCGGGGCACCGTCGCCGCCCCCGTCCCGCATCGCTGCGTGACGGGCGGCGGGAA

CAACACCCGGCGCGGTGGGCGCCAAGGAACAGTGCTGTCGGAGAGCGTCGCGCGTCGGTT

TCGACGCGCAGCGCGATCCTTCCATACGTCGAACCTTTACGACTCTCGGCAACGGATATC

TTGGCTCTCGCATCGATGAAGAACGTAGCGAAATGCGATACTTGGTGTGAATTGCAGAAT

CCCGTGAACCATCGAGTCTTTGAACGCAAGTTGCGCCCGAGGCTTTCCGGCCGAGGGCAC

GCCTGCCTGGGCGTCATGCCTCGCGTCGCTCCGTGCACCCTGCCCCTCGTCCCGGGGAGG

CAGCGGGAGCGGATGCGAAGATTGGCCCCCCGTGCCTGACGGCACGGCGGGTCGAAGTGC

GCGCCGCCGGTCGGGACGGACGTGGCGAGTGGTGGACGGACACGTACGACGCTGAGCGTC

GCCTCCGCCCCCCGGCCCCAGCGGTGCATGCAAGGAACCCACGCCGAGCACCCCTCGGAA

CACGACCCCAGGTCAG

>ITS_KF671243.1_Ophiopogon_c

CGAAACCGTAAACGCTCCTGCAGGGGCGGAGGGAGGGCGGATATTCAGACCGTCCGACCA

CGCACCTCGGGGCACCGTCGCCGCCCCCGTCCCGCATCGCTGCGTGACGGGCGGCGGGAA

CAACACCCGGCGCGGTGGGCGCCAAGGAACAGTGCTGTCGGAGAGCGCCGCGCGCCGGTT

TCGACGCGTAGCGCGATCCTTCCATACGTCGAACCTTTACGACTCTCGGCAACGGATATC

TTGGCTCTCGCATCGATGAAGAACGCAGCGAAATGCGATACTTGGTGTGAATTGCAGAAT

CCCGTGAACCATCGAGTCTTTGAACGCAAGTTGCGCCCGAGGCCATCCGGCCGAGGGCAC

GCCTGCCTGGGCGTCATGCCTCGCGTCGCTCCGTGCACCCTGCCCCTCGTCCCGGGGAGG

CAGCGGGAGCGGATGCGGAGATTGGCCCCCCGTGCTTGACGGCACGGCGGGTCGAAGTGC

GTGCCGCCGGTCGGGACGGACGTGGCGAGTGGTGGACGGACACGTACGGCGCTGAGCGTC

GCCTCCGCCCCTCGGCCCCAGCGGTGCATGCAAGGAACCCATGCCG

>ITS_KF671240.1_Ophiopogon_b

CGAACCCGTAAACGCTCCTGCAGGGGCGGAGGGAGGGCGGATATTCAGACCGTCCGACCA

CGCACCTCGGGGCACCATGGCCGCCCTCGCCTCGCATCGTTGCGTGACGGACGGCGGGAA

CAACACCCGGCGCGGTGGGCGCCAAGGAACAGTGCTGTCGGAGAGCGTCGCGCGCCGGTT

TCGGCGCGTAGTGCCATCCTTCCATACGTCGAACCTTTACGACTCTCGGCAACGGATATC

TTGGCTCTCGCATCGATGAAGAACGCAGCGAAATGCGATACTTGGTGTGAATTGCAGAAT

CCCGTGAACCATCGAGTCTTTGAACGCAAGTTGCGCCCGAGGCTATCCGGCCGAGGGCAC

GCCTGCCTGGGCGTCATGCCTCGCGTCGCTCCGTGCACCTTGCCCCTCGTCCCGGGGAGG

CAGCGGGAGCGGATGCGGAGATTGGCCCCCCGTGCCTTGACGGCGCGGCGGGTCGAAGTG

CGTGCCGCCGGTCGGGACGGACGCGGCGAGTGGTGGACGGACGTACGGCGCTGAACGTCG

CCTCCGCCCCCCGGCCCCAGCGGTGCATGCAAGGAACCCATGCCGAGCACCCCTCGGAAC

ACGACCCCAGGTCAG

>ITS_KF671233.1_Ophiopogon_b

CGAACCCGTAAACGCTCCTGCAGGGGCGGAGGGAGGGCGGATATTCAGACCGTCCGACCA

CGCACCTCGGGGCACCATGGCCGCCTTCGCCCCGCATCGCTGGTGACGGACGGCGGGAAC

AACACCCGGCGCGGTGGGCGCCAAGGAACAGTGCTGTCGGAGAGCGTCGCGCGCCGGTTT

CGGCGCGTAGTGCCATCCTTCCATACGTCGAACCTTTACGACTCTCGGCAACGGATATCT

TGGCTCTCGCATCGATGAAGAACGCAGCGAAATGCGATACTTGGTGTGAATTGCAGAATC

CCGTGAACCATCGAGTCTTTGAACGCAAGTTGCGCCCGAGGCTATCCGGCCGAGGGCACG

CCTGCCTGGGCGTCATGCCTCGCGTCGCTCCGTGCACCCTGCCCCTCGTCCCGGGGAGGC

AGCGGGAGCGGATGCGGAGATTGGCCCCCCGTGCCCGACGGCGCGGCGGGTCGAAGTGCG

TGCCGCCGGTCGGGACGGACGCGGCGAGTGGTGGACGGACACGTACGGCGCTGAACGTCG

CCTCCGCCCCCCGGCCCCAGCGGTGCATGCAAGGAACCCATGCCGAGCACCCCTCGGAAC

ACGACCCCAGGTCAG

>ITS_KF671238.1_Ophiopogon_b

CGAACCCGTAAACGCTCCTGCAGGGGCGGAGGGAGGGCGGATATTCAGACCGTCCGACCA

CGCACCTCGGGGCACCATGGCCGCCCTCGCCTCGCATCGCTGCGTGACGGACGGCGGGAA

CAACACCCGGCGCGGTGGGCGCCAAGGAACAGTGCTGTCGGAGAGCGTCGCGCGCCGGTT

TCGGCGCGCAGTGCCATCCTTCCATACGTCGAACCTTTACGACTCTCGGCAACGGATATC

TTGGCTCTCGCATCGATGAAGAACGTAGCGAAATGCGATACTTGGTGTGAATTGCAGAAT

CCCGTGAACCATCGAGTTTTTGAACGCAAGTTGCGCCCGAGGCTATCCGGCCGAGGGCAC

GCCTGCCTGGGCGTCATGCCTCGCGTCGCTCCGTTCACCTTGCCCCTCGTCCCGGGGAGG

CAGCGGGAGCGGATGCGGAGATTGGCCCCCCGTGCCTTGACGGCGCGGCGGGTTGAAGTG

CGTGCCGCCGGTCGGGACGGACGCGGCGAGTGGTGGACGGACACGTACGGCGCTGAACGT

CGCCTCCGCACCCCGGCCCCAGCGGTGCATGCAAGGAACCCATGCCGAGCACCCCTCGGA

ACACGACCCCAGGTCAG

>ITS_KF671235.1_Ophiopogon_b

CGAACCCGTAAACGCTCCTGCAGGGGCGGAGGGAGGGCGGATATTCAGACCGTCCGACCA

CGCACCTCGGGGCACCATGGCCGCCCTCGCCTCGCATCGCTGCGTGACGGACGGCGGGAA

CAACACCCGGCGCGGTGGGCGCCAAGGAACAGTGCTGTCGGAGAGCGTCGCGCGCCGGTT

TCGGCGCGTAGTGCCATCCTTCCATACGTCGAACCTTTACGACTCTCGGCAACGGATATC

TTGGCTCTCGCATCGATGAAGAACGTAGCGAAATGCGATACTTGGTGTGAATTGCAGAAT

CCCGTGAACCATCGAGTCTTTGAACGCAAGTTGCGCCCGAGGCTATCCGGTCGAGGGCAC

GCCTGCCTGGGCGTCATGCCTCGCGTCGCTCCGTGCACCTTGCCCCTCGTCCCGGGGAGG

CAGCGGGAGCGGATGCGGAGATTGGCCCCCCGTGCCTTGACGGCGCGGCGGGTCGAAGTG

CGTGCCGCCGGTCGGGACGGACGCGGCGAGTGGTGGACGGACGTACGGCGCTGAACGTCG

CCTCCGCCCCCCGGCCCCAGCGGTGCATGCAAGGAACCCATGCCGAGCACCCCTCGGAAC

ACGACCCCAGGTCAG

>ITS_KF671232.1_Ophiopogon_b

CGAACCCGTAAACGCTCCTGCAGGGGCGGAGGGAGGGCGGATATTCAGACCGTCCGACCA

CGCACCTCGGGGCACCATGGCCGCCTTCGCCTCGCATCGCTGCGTGACGGACGGCGGGAA

CAACACCCGGCGCGGTGGGCGCCAAGGAACAGTGCTGTCGGAGAGCGTCGCGCGCCGATT

TCGGCGCGTAGTGCCATCCTTCCATACGTCGAACCTTTATGACTCTCGGCAACGGATATC

TTGGCTCTCGCATCGATGAAGAACGTAGCGAAATGCGATACTTGGTGTGAATTGCAGAAT

CCCGTGAACCATCGAGTCTTTGAACGCAAGTTGCGCCCGAGGCTATCCGGCCGAGGGCAC

GCCTGCCTGGGCGTCATGCCTCGCGTCGCTCCGTGCACCCTGCCCCTCGTCCCGGGGAGG

CAGCGGGAGCGGATGCGGAGATTGGCCCCCCGTGCCCGACGGCGCGGCGGGTCGAAGTGC

GTGCCGCCGGTCGGGACGGACGCGGCGAGTGGTGGACGGACACGTACGGCGCTGAACGTC

GCCTCCGCCCCCCGGCCCCAGCGGTGCATGCAAGGAACCCATGCCGAGCACCCCTCGGAA

CACGACCCCAGGTCAG

>ITS_KC798477.1_Ophiopogon_pl

CGAACCCGTAAACGCTCCTGCAGGGGCGGAGGGAAGGCGGATATTCAGACCGTCCGACCA

CGCCCCTCGGGGCACCATGGCCGCCTTCGCCTCGCATCGCTGCGTGACGGACGGCGGGAA

CAACACCCGGCGCGGTGGGCGCCAAGGAACAGTGCTGTCGGAGAGCGTCGCGCGCCGATT

TCGGCGCGTAGTGCCATCCTTCCATACGTCGAACCTTTACGACTCTCGGCAACGGATATC

TTGGCTCTCGCATCGATGAAGAACGTAGCGAAATGCGATACTTGGTGTGAATTGCAGAAT

CCCGTGAACCATCGAGTCTTTGAACGCAAGTTGCGCCCGAGGCTATCCGGCCGAGGGCAC

GCCTGCCTGGGCGTCATGCCTCGCGTCGCTCCGTGCACCCTGCCCCTCGTCCCGGGGAGG

CAGCGGGAGCGGATGCGGAGATTGGCCCCCCGTGCCCGACGGCGCGGCGGGTCGAAGTGC

GTGCCGCCGGTCGGGACGGACGCGGCGAGTGGTGGACGGACACGTACGGCGCCGAACGTC

GCCTCCGCCCCCCGGCCCCAGCGGTGCATGCAAGGAACCCATGCCGAGCACCCCTCGGAA

CACGACCCCAGGTCAG

>Lir_628_1259_EM_ITS_ITS1

AAGGCGGATATTCAGACCGTCCGACCACGCTCCTCGGGGCACCATGGCCGCCTTCGCCTC

GCATCGCTGCGTGACGGACGGCGGGAACAACACCCGGCGCGGTGGGCGCCAAGGAACAGT

GCTGTCGGAGAGCGTCGCGCGCCGATTTCGGCGCGTAGTGCCATCCTTCCATACGTCGAA

CCTTTACGACTCTCGGCAACGGATATCTTGGCTCTCGCATCGATGAAGAACGTAGCGAAA

TGCGATACTTGGTGTGAATTGCAGAATCCCGTGAACCATCGAGTCTTTGAACGCAAGTTG

CGCCCGAGGCTATCCGGCCGAGGGCACGCCTGCCTGGGCGTCATGCCTCGCGTCGCTCCG

TGCACCCTGCCCCTCGTCCCGGGGAGGCAGCGGGAGCGGATGCGGAGATTGGCCCCCCGT

GCCCGACGGCGCGGCGGGTCGAAGTGCGTGCCGCCGGTCGGGACGGACGCGGCGAGTGGT

GGACGGACACGTACGGCGCCGAACGTCGCCTCCGCCCCCCGGCCCCAGCGGTGCATGCAA

GGAACCCATGCCGAGCACC

>Lir-677_1343_EM_ITS_ITS1

GGAAGGCGGATATTCAGACCGTCCGACCACGCTCCTCGGGGCACCATGGCCGCCTTCGCC

TCGCATCGCTGCGTGACGGACGGCGGGAACAACACCCGGCGCGGTGGGCGCCAAGGAACA

GTGCTGTCGGAGAGCGTCGCGCGCCGATTTCGGCGCGTAGTGCCATCCTTCCATACGTCG

AACCTTTACGACTCTCGGCAACGGATATCTTGGCTCTCGCATCGATGAAGAACGTAGCGA

AATGCGATACTTGGTGTGAATTGCAGAATCCCGTGAACCATCGAGTCTTTGAACGCAAGT

TGCGCCCGAGGCTATCCGGCCGAGGGCACGCCTGCCTGGGCGTCATGCCTCGCGTCGCTC

CGTGCACCCTGCCCCTCGTCCCGGGGAGGCAGCGGGAGCGGATGCGGAGATTGGCCCCCC

GTGCCCGACGGCGCGGCGGGTCGAAGTGCGTGCCGCCGGTCGGGACGGACGCGGCGAGTG

GTGGACGGACACGTACGGCGCCGAACGTCGCCTCCGCCCCCCGGCCCCAGCGGTGCATGC

>Ophio_697_1367_EM_ITS_ITS1

CACCATGGCCGCCTTCGCCTCGCATCGCTGCGTGACGGACGGCGGGAACAACACCCGGCG

CGGTGGGCGCCAAGGAACAGTGCTGTCGGAGAGCGTCGCGCGCCGATTTCGGCGCGTAGT

GCCATCCTTCCATACGTCGAACCTTTACGACTCTCGGCAACGGATATCTTGGCTCTCGCA

TCGATAAAGAACGTAGCGAAATGCGATACTTGGTGTGAATTGCAGAATCCCGTGAACCAT

CGAGTCTTTGAACGCAAGTTGCGCCCGAGGCTATCCGGCCGAGGGCACGCCTGCCTGGGC

GTCATGCCTCGCGTCGCTCCGTGCACCCTGCCCCTCGTCCCGGGAAGGCAGCGGGAGCGG

ATGCGGAGATTGGCCCCCCGTGCCCGACGGCGCGGCGGGTCGAAGTGCGTGCCGCCTGTC

GGGACGGACGCGGCGAGTGGTGGACGGACACGTACGGCGCCAAAAGTCGCCTCCGCCCCC

CGGCCCCAGCGGTGCATGC

>Opjhio_698_1368_EM_ITS_ITS1

TTTCGGCGCGTAGTGCCATCCTTCCATACGTCGAACCTTTACGACTCTCGGCAACGGATA

TCTTGGCTCTCGCATCGATAAAGAACGTAGCGAAATGCGATACTTGGTGTGAATTGCAGA

ATCCCGTGAACCATCGAGTCTTTGAACGCAAGTTGCGCCCGAGGCTATCCGGCCGAGGGC

ACGCCTGCCTGGGCGTCATGCCTCGCGTCGCTCCGTGCACCCTGCCCCTCGTCCCGGGGA

GGCAGCGGGAGCGGATGCGGAGATTGGCCCCCCGTGCCCGACGGCGCGGCGGGTCGAAGT

GCGTGCCGCCTGTCGGGACGGACGCGGCGAGTGGTGGACGGACACGTACGGCGCCAAACG

TCGCCTCCGCCCCCCGGCCCCAGCGGTGCATGCAAGGAA

>Ophio_702_1372_EM_ITS_ITS1

CATCGCTTCGTGACGGACGGCGGGAACAACACCCGGCGCGGTGGGCGCCAAGGAACAGTG

CTGTCGGAGAGCGTCGCGCGCCGATTTCGGCGCGTAGTGCCATCCTTCCATACGTCGAAC

CTTTACGACTCTCGGCAACGGATATCTTGGCTCTCGCATCGATGAAGAACGTAGCGGAGT

GCGATACTTGGTGTGAATTGCAGAATCCCGTGAACCATCGAGTCTTTGAACGCAAGTTGC

GCCCGAGGCTATCCGGCCGAGGGCACGCCTGCCTGGGCGTCATGCCTCGCGTCGCTTCGT

GCACCCTGCCCCTCGTCCCGGGGAGGCAGCGGGAGCGGATGCGGAGATTGGCCCCCCGTG

CCCGACGGCGCGGCGGGTCGAAGTGCGTGCCGCCTGTYGGGACGGACGCGGCGAGTGGTG

GACGGACACGTACGGCGCCBAACGTCGCCTCCGSCCCCCGGCCCCAGCGGTKCATGCCAG

>Ophio_700_1370_EM_ITS_ITS1

CCTCGCATCGCTTCGTGACGGACGGCGGGAACAACACCCGGCGCGGTGGGCGCCAAGGAA

CAGTGCTGTCGGAGAGCGTCGCGCGCCGATTTCGGCGCGTAGTGCCATCCTTCCATACAT

CGAACCTTTACGACTCTCGGCAACGGATATATGGGCTCTCGCATCGCTAAAGAACGTACC

GGAGTGCGATACTTGGTGTGAATTGCAAAATCCCGTGGACCATCGCGTCTTTGAACGCAA

GTGGCGCCCGAGGCTATCCGGCCGAGGGCACGCCTGCCTGGGCGTCATGCCTCGCGTCGC

TCCGTGCACCCTGCCCCTCGGCCCGGGAGGCCGCGGGAGCGGATGCGGAGATTGGCCCCC

CGTGCCCAACGGCGCGCCGGG

>Ophio_701_1371_EM_ITS_ITS1

TTCGCCTCGCATCGCTTCGTGACGGACGGCGGGAACAACACCCGGCGCGGTGGGCGCCAA

GGAACAGTGCTGTCGGAGAGCGTCGCGCGCCGATTTCGGCGCGTAGTGCCATCCTTCCAT

ACGTCGAACCTTTACGACTCTCGGCAACGGATATCTTGGCTCTCGCATCGATGAAGAACG

TACCGGAGTGCGATACTTGGTGTGAATTGCAGAATCCCGTGAACCATCGAGTCTTTGAAC

GCAAGTTGCGCCCGAGGCTATCCGGCCGAGGGCACGCCTGCCTGGGCGTCATGCCTCGCG

TCGCTCCGTGCACCCTGCCCCTCGTCCCGGGGAGGCAGCGGGAGCGGATGCGGAGATTGG

CCCCCCGTGCCCGACGGCGCGSCGGGTCGAAGTGCGTGCCGCCTGTCGGGACGGACGCGG

CGAGTGGTGGACGGACACGTA

>Ophio_704_1374_EM_ITS_ITS1

CATGGCCGCCTTCGCCTCGCATCGCTTCGTGACGGACGGCGGGAACAACACCCGGCGCGG

TGGGCGCCAAGGAACAGTGCTGTCGGAGAGCGTCGCGCGCCGATTTCGGCGCGTAGTGCC

ATCCTTCCATACGTCGAACCTTTATGACTCTCGGCCACGGATATATTGGCTCTCGCATCG

CTGAAGAACGTACCGGAGTGCGATACTTGGTGTGAATTGCAGAATCCCGTGAACCATCGC

GTCTTTGAACGCAAGTGGCGCCCGAGGCTATCCGGCCGAGGGCACGCCTGCCTGGGCGTC

ATGCCTCGCGTCGCTTCGTGCACCCTGCCCCTCGGCCCGGGAGGCAGCGGGAGCGGATGC

GGAGATTGGCCCCCCGTGCCCGACGGCGCGCCSGGTCGAAGTGCGTGCCGCCCGTCGGGA

CGGACGCGCCAAGTKGTGGA

>Ophio_703_1373_EM_ITS_ITS1

CCATGGCCGCCTTCGCCTCGCATCGCTTCGTGACGGACGGCGGGAACAACACCCGGCGCG

GTGGGCGCCAAGGAACAGTGCTGTCGGAGAGCGTCGCGCGCCGATTTCGGCGCGTAGTGC

CATCCTTCCATACGTCGAACCTTTATGACTCTCGGCAACGGATATATTGGCTCTCGCATC

GATGAAGAACGTACCGAAGTGCGATACTTGGTGTGAATTGCAGAATCCCGTGAACCATCG

CGTCTTTGAACGCAAGTGGCGCCCGAGGCTATCCGGCCGAGGGCACGCCTGCCTGGGCGT

CATGCCTCGCGTCGCTCCGTGCACCCTGCCCCTCGTCCCGGGGAGGCAGCGGGAGCGGAT

GCGGAGATTGGCCCCCCGTGCCCGACGGCGCGCSGGTCGAAGTGCGTGCCGCCTGTAGGG

>Ophio_680_ITS1

TCGCATCGCTGCGTGACGGACGGCGGGAACAACACCCGGCGCGGTGGGCGCCAAGGAACA

GTGCTGTCGGAGAGCGTCGCGCGCCGATTTCGGCGCGTATTGCCATCCTTCCATACGTCG

AACCTTTATGACTCTCGGCAACGGATATCTTGGCTCTCGCATCGATGAAGAACGTACCGA

AATGCGATACTTGGTGTGAATTGCAGAATCCCGTGAACCATCGAGTCTTTGAACGCAAGT

TGCGCCCGAGGCTATCCGGCCGAGGGCACGCCTGCCTGGGCGTCATGCCTCGCGTCGCTC

CGTGCACCCTGCCCCTCGTCCCGGGGAGGCAGCGGGAGCGGATGCGGAGATTGGCCCCCC

GTGCCCGACGGCGCGGCGGGTCGAAGTGCGTGCCGCCGGTCGGGACGGACGCGGCGAGTG

GTGGACGGACACGTACGGC

>Ophio_678_1353_EM_ITS_ITS1

CCGACCACGCACCTCGGGGCACCATGGCCGCCTTCGCCTCGCATCGCTGCGTGACGGACG

GCGGGAACAACACCCGGCGCGGTGGGCGCCAAGGAACAGTGCTGTCGGAGAGCGTCGCGC

GCCGATTTCGGCGCGTAGGGCCATCCTTCCATACGTCGAACTTTATGACTCTCGGCAACG

GATATCTTGGCTCTCGCATCGATAAGAACGTAGCGAAATGCGATACTTGGTGTGAATTGC

AGAATCCCGTGAACCATCGAGTCTTTGAACGCAAGTTGCGCCCGAGGCTATCCGGCCGAG

GGCACGCCTGCCTGGGCGTCATGCCTCGCGTCGCTCCGTGCACCCTGCCCCTCGTCCCGG

GGAGGCAGCGGGAGCGGATGCGGAGATTGGCCCCCCGTGCCCGACGGCGCGCGGGTCGAA

GTGCGTGCCGCCGGTCGGGACGGACGCGGCGAGTGGTGGACGGACACGTACGGCGCTAAA

CGTCGCCTCCGCCCCCCGGCCCCAGCGGTGCAKGCAAGGAA

>Ophio_686_1360_EM_ITS_ITS1

ATGGCCGCCTTCGCCTCGCATCGCTGCGTGACGGACGGCGGGAACAACACCCGGCGCGGT

GGGCGCCAAGGAACAGTGCTGTCGGAGAGCGTCGCGCGCCGATTTCGGCGCGTAGGGCCA

TCCTTCCATACGTCGAACCTTTATGACTCTCGGCAACGGATATCTTGGCTCTCGCATCGA

TAAGAACGTAGCGAATGCGATACTTGGTGTGAATTGCAGAATCCCGTGAACCATCGAGTC

TTTGAACGCAAGTTGCGCCCGAGGCTATCCGGCCGAGGGCACGCCTGCCTGGGCGTCATG

CCTCGCGTCGCTCCGTGCACCCTGCCCCTCGTCCCGGGGAGGCAGCGGGAGCGGATGCGG

AGATTGGCCCCCCGTGCCCGACGGCGCGGCGGGTCGAAGTGCGTGCCGCCGGTCGGGACG

GACGCGGCGAGTGGTGGACGGACACGTACGGCGCTAAACGTCGCCTCCGCCCCCCGGCCC

CAGCGGTGCATGCAAGGAACC

>Ophio_699_1369_EM_ITS_ITS1

GAAGAACGTAGCGAAATGCGATACTTGGTGTGAATTGCAGAATCCCGTGAACCATCGAGT

CTTTGAACGCAAGTTGCGCCCGAGGCTATCCGGCCGAGGGCACGCCTGCCTGGGCGTCAT

GCCTCGCGTCGCTCCGTGCACCCTGCCCCTCGTCCCGGGGAGGCAGCGGGAGCGGATGCG

GAGATTGGCCCCCCGTGCCCGACGGCGCGGCGGGTCGAAGTGCGTGCCGCCGGTCGGGAC

GGACGCGGCGAGTGGTGGACGGACACGTACGGCGCCRAACGTCGCCTCCGCCCCCCGGCC

CCAGCGGTGCATGCAAGGAAC

>Ophio_690_1362_EM_ITS_ITS1

TCCTTCCATACGTCGAACCTTTACGACTCTGGGCAACGGATATCTTGGCTCTCGCATCGA

TTAAGAACGTAGCGAAATGCGATACTTGGTGTGAATTGCAGAATCCCGTGAACCATCGAG

TCTTTGAACGCAAGTTGCGCCCGAGGCTATCCGGCCGAGGGCACGCCTGCCTGGGCGTCA

TGCCTCGCGTCGCTTCGTGCACCCTGCCCCTCGTCCCGGGGAGGCAGCGGGAGCGGATGC

GGAGATTGGCCCCCCGTGCCCGACGGCGCGTCGGGTCGAAGTGCGTGCCGCCCGTCGGGA

CGGACGCGGCGAGTGGTGGACGGACACGTACGGCGCCAAACGTCGCCTCCGCCCCCCGGC

CCCTGCGGTGCATGCTAGGAA

>ITS_KF671269.1_Ophiopogon_i

CGAACCCGTAAACGCTCCTGCAGGGGCGGAGGGAGGGCGGATATTCTGACCGCCTGACCT

CGCCCCTCGGGGCACCATGGCTGCCCCCGCCTCGCATCGCTGCGGGACGGGCGGCGGGAA

CAACACCCGGCGCGGTGGGCGCCAAGGAACAGTGCTGACGGAGAGCGCCGCGCGCCGGTC

TCGGCGCGCAGCGCGATCCCTCCATACGTCGAACTCTTACGACTCTCGGCAACGGATATC

TTGGCTCTCGCATCGATGAAGAACGTAGCGAAATGCGATACTTGGTGTGAATTGCAGAAT

CCCGTGAACCATCGAGTCTTTGAACGCAAGTTGCGCCCGAGGCTATCCGGCCGAGGGCAC

GCCTGCCTGGGCGTCACGCCTAGCGTCGCTCCGCGCACCTCGCCTCCTCGTCACGGGGGG

CGGCGGGTGCGGATGCGGAGATTGGCCCCCCGTGCCTGAAGGCGCGGCGGGTCGAAGTGC

GACCGCCGGTCGGGACGGACGCGGCGAGTGGTGGACGGACACGCACGGCGCTGAACGTCG

CCTCCGCCCCCGGCCCCAGCGGTGCATGCAAGGAACCCATGCCGAGCACCCCTCGGAACA

CGACCCCCGTCAG

>ITS_KF671270.1_Ophiopogon_i

CGAACCCGTAAACGCTCCTGCAGGGGCGGAGGGAGGGCGGATATTCTGACCGCCTGACCT

TCGCCCCTCGGGGCACCATGGCTGCCCCCGCCTCGCATCGCTGCGGGACGGGCGGCGGGA

ACAACACCCGGCGCGGTGGGCGCCAAGGAACAGTGCTGACGGAGAGCGCCGCGCGCCGGT

CTCGGCGCGCAGCGCGATCCCTCCATACGTCGAACTCTTACGACTCTCGGCAACGGATAT

CTTGGCTCTCGCATCGATGAAGAACGTAGCGAAATGCGATACTTGGTGTGAATTGCAGAA

TCCCGTGAACCATCGAGTCTTTGAACGCAAGTTGCGCCCGAGGCTATCCGGCCGAGGGCA

CGCCTGCCTGGGCGTCACGCCTAGCGTCGCTCCGCGCACCTCGCCCCCTCGTCACGGGGG

ACGGCGGGTGCGGATGCGGAGATTGGCCCCCCGTGCCTGAAGGCGCGGCGGGTCGAAGTG

CGACCGCCGGTCGGGACGGACGCGGCGAGTGGTGGACGGACACGCACGGCGCTGAACGTC

GCCTCCGCCCCCGGCCCCAGCGGTGCATGCAAGGAACCCATGCCGAGCACCCCTCGGAAC

ACGACCCCAGGTCAG

>ITS_KF671271.1_Ophiopogon_i

CGAACCCGTAAACGCTCCTGCAGGGGCGGAGGGAGGGCGGATATTCTGACCGCCTGACCT

TCCCCCTCGGGGCACCATGGCTGCCCCCCCCTCGCATCGCTGCGGGACGGGCGGCGGGAA

CAACACCCGGCGCGGTGGGCGCCAAGGAACACTGCTGACGGAGAGCGCCGCGCGCCGGTC

TCGGGGCGCACCGCGATCCCTCCATACGTCGAACTCTTACGACTCTCGGCAACGGATATC

TTGGCTCTCGCATCGATGAAGAACGTAGCGAAATGCGATACTTGGTGTGAATTGCAGAAT

CCCGTGAACCATCGAGTCTTTGAAAGCAAGTTGCGCCCGAGGCTATCCGGCCGAGGGCAC

GCCTGCCTGGGCGTCACGCCTAGAGTCGCTCCGCGCACCTCGCCCCCTCGTCTCGGGGGG

CGGCGGGTGCGGATGCGGAAATTGGCCCCCCGTGCCTGAAGGCGCGGCGGGTCAAAGTGC

GACCGCCGGTCGGGACGGACGCGGCGAGTGGTGGACGGACACGCACGGCGCTGAACGTCG

CCTCCGCCCCCGGCCCCAGCGGTGCATGCAAGGAACCCATGCCGAGCACCCCTCGGAACA

CGACCCCGGCA

>Ophio_685_1359_EM_ITS5p_ITS5p

ACGCTCCTGCAGGGGCGGAGGGAGGGGAACGGATATTCCGTTCCGGTCGCCCGACCTCGC

ACCTCGGGGCACCATGGCCGTCCCTGCCCCGCATCGCTGCGGGACGGGCTGCGGGAACAA

CACCCGGCGCGGTGGGCGCCAAGGAATAGTGCTGTCGGAGAGCGCCGTGTGCCGGTCTGG

GCGCGCGGCGCGATCCTTCCATACGTCGAACCTTTACGACTCTCGGCAACGGATATCTTG

GCTCTCGCATCGATGAAGAACGTAGCGAAATGCGATACTTGGTGTGAATTGCAGAATCCC

GTGAACCATCGAGTCTTTGAACGCAAGTTGCGCCCGAGGCTATCCGGCCGAGGGCACGCC

TGCCTGGGCGTCATGCCTCGCGTCGCTCCGTGCACCCCGCCTCTCGTCCCTGGGAGGCGG

CGGGAGCGGATGCGGAGATTGGCCCCCCGTGCCTGACGGCGCGGCGGGCCGAAGTGCGTG

CCGCCGGTCGGGTCGGACGCGGCGAGTGGTGGACGGACTCG

>Lir_667_1335_EM_ITS5p_ITS5p

GGCGGAGGGAGGGGAACGGATATTCCGTTCCGGTCGCCCGACCTCGCACCTCGGGGCACC

ATGGCCGTCCCTGCCCCGCATCGCTTCGGGACGGGCTGCGGGAACAACACCCGGCGCGGT

GGGCGCCAAGGAATAGTGCTGTCGGAGAGCGCCGTGTGCCGGTCTGGGCGCGCGGCGCGA

TCCTTCCATACGTCGAACCTTTACGACTCTCGGCAACGGATATCTTGGCTCTCGCATCGA

TGAAGAACGTAGCGAAATGCGATACTTGGTGTGAATTGCAGAATCCCGTGAACCATCGAG

TCTTTGAACGCAAGTTGCGCCCGAGGCTATCCGGCCGAGGGCACGCCTGCCTGGGCGTCA

TGCCTCGCGTCGCTCCATGCACCCCGCCTCTCGTCCCTGGGAGGCGGCGGGAGCGGATGC

GGAGATTGGCCCCCCGTGCCTGACGGCACGACGGGCCGAAGTGCGTGCCGCCGGTCGGGT

CGGACGCGGCGAGTGGTGGA

>ITS_KX231372.1_Ophiopogon_l

GCATCGCTGCGGGACGGGCGGCGGGAACAACACCCGGCGCGGTGGGCGCCAAGGAACAGT

GCTGTAGGAGAGCGCGTAGCGCGGTCCTTCCATACGTCGAACCTTTACGACTCTCGGCAA

CGGATATCTTGGCTCTCGCATCGATGAAGAACGTAGCGAAATGCGATACTTGGTGTGAAT

TGCAGAATCCCGTGAACCATCGAGTCTTTGAACGCAAGTTGCGCCCGAGGCTATCCGGCC

GAGGGCACGCCTGCCTGGGCGTCATGCCTCGCGTCGCTCCGTGAACCCCGACCCCCCGTC

CCGGGGAGGCGGCGGGTGCGGATGCGGAGATTGGCCCCCCGTGCCTGACGGCGTGGCGGG

TCGAAGTGCGTGCCGCCGGTCGGGACGGACGCGGCGAGTGGTGGACGGACACGTACGGCG

CTGAACGTCGCCTCCGACCCCCGGCCCCATCGGTGCATGCAAGGAACCCACGCCGAGCAC

C

>ITS_KX231369.1_Ophiopogon_l

CGAACCCGTAAACGCTCCTGCAGGGGCGGAGGGGAAGGCGGATATTCCGACCGCTCGACC

TCGCACCTCGGGGCACCATGGCCGCCCCCGCCCCGCATCGCTGCGGGACGGGCGGCGGGA

ACAACACCCGGCGCGGTGGGCGCCAAGGAACAGTGCTGTAGGAGAGCGCGTAGCGCGGTC

CTTCCATACGTCGAACCTTTACGACTCTCGGCAACGGATATCTTGGCTCTCGCATCGATG

AAGAACGTAGCGAAATGCGATACTTGGTGTGAATTGCAGAATCCCGTGAACCATCGAGTC

TTTGAACGCAAGTTGCGCCCGAGGCTATCCGGCCGAGGGCACGCCTGCCTGGGCGTCATG

CCTCGCGTCGCTCCGTGAACCCCGACCCCCCGTCCCGGGGAGGCGGCGGGTGCGGATGCG

GAGATTGGCCCCCCGTGCCTGACGGCGTGGCGGGTCGAAGTGCGTGCCGCCGGTCGGGAC

GGACGCGGCGAGTGGTGGACGGACACGTACGGCGCTGAACGTCGCCTCCGACCCCCGGCC

CCATCGGTGCATGCAAGGAACCCACGCCGAGCACC

>Ophio_691_1363_EM_ITS_ITS1

GCACCTTGCCCCCCTCCTCCCCGCATTTCTCCGGGACGGGCGGCGGGAACAACACCCGGC

GCGATGGGCGCCAAGGAACTGTGCTTTGTCGGAGAGCGTCGCGTGTCGGTGTCGGCGCGG

AGCGTGTTCCTTCCATACATCCAATCTTTCCCACGCTCGTCGACGGATATGTTGGCTCTT

GCATCGATAAAGAAGGTAACTGACTGCGATACTTGGTGTGAATTGCACAAGCCCGTGAAC

CATCGTGTCTTTGAACGCAAGTCGCGCCBGAGGCTATCCGGCCGAGGGCACGCCTGCCTG

GGCGTCACGCCTCGTGTCGCTGCGTGCACCCCGTCCCGAGTAGGCGGCGGGTGCGGATGC

GTAGATTGGCCCCCCGTGCCTGACGGCACGTCGGGTCGAATTGCGTATCGCCGGACGGGA

CGGACGCGTCGAGTGGTGAACGGACACGTACGGCGCTTAACGTCKCCTCCGCCCCCCGGC

>Ophio_683_1357_EM_ITS_ITS1

GGGGTGGAAACAACACCCGGTGCGATGGGCGCCAAAGAACTGTGCTTTGTCAGAGACCGT

CGCGTGTCTGTGTCGGCACGCAGCGTGTTCCTTCCATACAACCAATCTTTCCCACGCTAA

TCGACAGAAATGTTGTCTCTTGCATCAATAAAGAACGTAACTGACTGACATACTGGGTGT

GAATTGCACATGCCCATGAACCAGCCTGTCTTTGAACGCAAGTCGCGACGGTGGCTATCC

AGCCGAGGGCACGCCTGCCTGGGCGTCACGCCGCGTGTCGCTGCGTGCACCCCGTCCCGA

GTAGGCGGCGGGTGCGCATACGTAGATCGGCCCCCCGTGCCTGACGGCGCGTCGGGTCGA

ATTGCGTACCGCCGGACGG

>Ophio_684_1358_EM_ITS5pM_ITS5p

GGCCGCCCCCGCCCCGCATTGCTGCGGGACGGGCGGCGGGAACAACACCCGGCGCGGTGG

GCGCCAAGGAACAGTGCTTGTCGGAGAGCGTCGCGTGCCGGTCTCGGCGCGCAGCGTGAT

CCTTCCATACGTCGAACCTTTACGACTCTCGGCAACGGATATCTTGGCTCTCGCATCGAT

GAAGAACGTAGCGAAATGCGATACTTGGTGTGAATTGCAGAATCCCGTGAACCATCGAGT

CTTTGAACGCACGTTGCGCCCGAGGCTATCCGGCCGAGGGCACGCCTGCCTGGGCGTCAC

GCCTCGCGTCGCTCCGTGCACCCCGTCCCGAGGAGGCGGCGGGTGCGGATGCGGAGATTG

GCCCCCCGTGCCTGACGGCACGGCGGGTCGAAGTGCGTACCGCCCGTCGGGACGGACGCG

GCGAGTGGTGGA

>Ophio_692_1364_EM_ITS5p_ITS5p

CGTAAACGCTCCTGCAGGGGCGGAGGGAGGGCGCGGATATTCCGACCGCCCGATCTCGGT

ACCGCGGGGCACCATGGCCGCCCCCGCCCCGCATTGCTGCGGGACGGGCGGCGGGAACAG

CACCCGGGKGGATGGGCGCCAAGGAACAATGCTTTGTCGGAGAGCGTCGCGTGCCGGTCT

CGGCGCGCAGCGTGATCCTTCCATACGTCGAACCTTTACGACTCTCGGCAACGGATATCT

TGGCTCTCGCATCGATGAAGAACGTAGCGAAATGCGATACTTGGTGTGAATTGCAGAATC

CCGTGAACCATCGAGTCTTTGAACGCAAGTTGCGCCCGAGGCTATCCGGCCGAGGGCACG

CCTGCCTGGGCGTCACGCCTCGCGTCGCTCCGTGCACCCCGTCCCGATGAGGCGGCGGGT

GCGGATGCGGAGATTGGCCCCCCGTGCCTGACGGCGCGGCGGGTCGAAGTGCGTACCGCC

GGTCGGGACGGACGCGGCGAGTGGTGGACGGACACGTACG

>Ophio_705_1375_EM_ITS5p_ITS5p

GCGGAGGGAGGGCGCGGATATTCCGACCGCCCGATCTCGGTACCGCGGGGCACCATGGCC

GCCCCCGCCCCGCATTGCTGCGGGACGGGCGGCGGGAACAACACCCGGCGCGATGGGCGC

CAAGGAACAGTGCTTTGTCGGAGAGCGTCGCGTGCCGGTCTCGGCGCGCAGCGTGATCCT

TCCATACGTCGAACCTTTACGACTCTCGGCAACGGATATCTTGGCTCTCGCATCGATGAA

GAACGTAGCGAAATGCGATACTTGGTGTGAATTGCAGAATCCCGTGAACCATCGAGTCTT

TGAACGCAAGTTGCGCCCGAGGCTATCCGGCCGAGGGCACGCCTGCCTGGGCGTCACGCC

TCGCGTCGCTCCGTGCACCCCGTCCCGATGAGGCGGCGGGTGCGGATGCGGAGATTGGCC

CCCCGTGCCTGACGGCGCGGCGGGTCGAAGTGCGTACCGCCGGTCGGGACGGACGCGGCG

AGTGGTGGACGGACACGTACG

>Ophio_688_1361_EM_ITS5p_ITS5p

GGAGGGAGGGCGCGGATATTCCGACCGCCCGATCTCGGTACCGCGGGGCACCATGGCCGC

CCCCGCCCCGCATTGCTGCGGGACGGGCGGCGGGAACAACACCCGGCGCGATGGGCGCCA

AGGAACAGTGCTTTGTCGGAGAGCGTCGCGTGCCGGTCTCGGCGCGCAGCGTGATCCTTC

CATACGTCGAACCTTTACGACTCTCGGCAACGGATATCTTGGCTCTCGCATCGATGAAGA

ACGTAGCGAAATGCGATACTTGGTGTGAATTGCAGAATCCCGTGAACCATCGAGTCTTTG

AACGCAAGTTGCGCCCGAGGCTATCCGGCCGAGGGCACGCCTGCCTGGGCGTCACGCCTC

GCGTCGCTCCGTGCACCCCGTCCCGATGAGGCGGCGGGTGCGGATGCGGAGATTGGCCCC

CCGTGCCTGACGGCGCGGCGGGTCGAAGTGCGTACCGCCGGTCGGGACGGACGCGGCGAG

TGGTGGACGGACACGTACGG

>Ophio_706_1376_EM_ITS5pM_ITS5p

GAGGGCGCGGATATTCCGACCGCCCGATCTCGGTACCGCGGGGCACCATGGCCGCCCCCG

CCCCGCATTGCTGCGGGACGGGCGGCGGGAACAACACCCGGCGCGATGGGCGCCAAGGAA

CAGTGCTTTGTCGGAGAGCGTCGCGTGCCGGTCTCGGCGCGCAGCGTGATCCTTCCATAC

GTCGAACCTTTACGACTCTCGGCAACGGATATCTTGGCTCTCGCATCGATAAAGAACGTA

GCGAAATGCGATACTTGGTGTGAATTGCAGAATCCCGTGAACCATCGAGTCTTTGAACGC

AAGTTGCGCCCGAGGCTATCCGGCCGAGGGCACGCCTGCCTGGGCGTCACGCCTCGCGTC

GCTCCGTGCACCCCGTCCCGAGGAGGCGGCGGGTGCGGATGCGGAGATTGGCCCCCCGTG

CCTGACGGCGCGGCGGGTCGAAGTGCGTACCGCCGGTCGGGACGGACGCGGCGAGTGGTG

GACGGACACGTACGGCGCT

>Ophio_708_1377_EM_ITS5p_ITS5p

GGGAGGGCGCGGATATTCCGACCGCCCGATCTCGGTACCGCGGGGCACCATGGCCGCCCC

CGCCCCGCATTGCTGCGGGACGGGCGGCGGGAACAACACCCGGCGCGATGGGCGCCAAGG

AACAGTGCTTTGTCGGAGAGCGTCGCGTGCCGGTCTCGGCGCGCAGCGTGATCCTTCCAT

ACGTCGAACCTTTACGACTCTCGGCAACGGATATCTTGGCTCTCGCATCGATGAAGAACG

TAGCGAAATGCGATACTTGGTGTGAATTGCAGAATCCCGTGAACCATCGAGTCTTTGAAC

GCAAGTTGCGCCCGAGGCTATCCGGCCGAGGGCACGCCTGCCTGGGCGTCACGCCTCGCG

TCGCTCCGTGCACCCCGTCCCGATGAGGCGGCGGGTGCGGATGCGGAGATTGGCCCCCCG

TGCCTGACGGCGCGGCGGGTCGAAGTGCGTACCGCCGGTCGGGACGGACGCGGCGAGTGG

TGGACGGACACGTACGGCGC

>Ophio_679_1354_EM_ITS_ITS1-1

CCGATCTCGTACCGCGGGGCACCATGGCCGCCCCCGCCCCGCATTGCTGCGGGACGGGCG

GCGGGAACAACACCCGGCGCGGTGGGCGCCAAGGAACAGTGCTTGTCGGAGAGCGTCGCG

TGCCGGTCTCGGCGCGCAACGTGATCCTTCCATGCGTCGAACCTTTACGACTCTCGGCAA

CGGATATCTTGGCTCTCGCATCGATAAAGAACGTAGCGAAATGCGATACTTGGTGTGAAT

TGCAGAATCCCGTGAACCATCGAGTCTTTGAACGCAAGTTGCGCCCGAGGCTATCCGGCC

GAGGGCACGCCTGCCTGGGCGTCACGCCTCGCGTCGCTCCGTGCACCCCGTCCCGAGGAG

GCGGCGGGTGCGGATGCGGAGATTGGCCCCCCGTGCCTGACGGCACGGCGGGTCGAAGTG

CGTACCGCCGGTCGGGACGGACGCGGCGAGTGGTGGACGGACACGTACGGCGCTGGACGT

CGCCTCCGCCCCCCGGCCACGGCGGCACATGCAAGGAACCC

>ITS_KF671259.1_Ophiopogon_j

CGAACTCGTAAACGCTCCTGCAGGGGCGGAGGGAGGGCGCGGATATTCCGACCGCCCGAT

CTCGGTACCGCGGGGCACCATGGCCGCCCCCGCCCCGCATTGCTGCGTGACGGGCGGCGG

GAACAACACCCGGCGCGATGGGCGCCAAGGAACAGTGCTTGTCGGAGAGCGTCGCGTGCC

GGTCTCGGCGCGCAGCGTGATCCTTCCATACGTCGAACTTTTACGACTCTCGGCAACGGA

TATCTTGGCTCTCGCATCGATGAAGAACGTAGCGAAATGCGATACTTGGTGTGAATTGCA

GAATCCCGTGAACCATCGAGTCTTTGAACGCAAGTTGCGCCCGAGGCTATCCGGCCGAGG

GCACGCCTGCCTGGGCGTCACGCCTCGCGTCGCTCCGTGCACCCCGTCCCGAGGAGGCGG

CGGGTGCGGATGCGGAGATTGGCCCCCCGTGCCTGACGGCACGGCGGGTCGAAGTGCGTA

CCGCCGGTCGGGACGGACGCGGCGAGTGGTGGACGGACACGTACGGCGCTGAACGTCGCC

TCCGCCCCCCGGCCACGGCGGTACATGCAAGGAACCCACGCCGAGCATCCCTCGGAACAC

GACCCCAGGTCAG

>ITS_KF671256.1_Ophiopogon_j

CGAACTCGTAAACGCTCCTGCAGGGGCGGAGGGAGGGCGCGGATATTCCGACCGCCCGAT

CTCGGTACCGCGGGGCACCATGGCCGCCCCCGCCCCGCATTGCTGCGGGACGGGCGGCGG

GAACAACACCCGGCGCGATGGGCGCCAAGGAACAATGCTTTGTCGGAGAGCGTCGCGTGC

CGGTCTCGGCGCGCAGCGTGATCCTTCCATACGTCGAACCTTTACGACTCTCGGCAACGG

ATATCTTGGCTCTCGCATCGATGAAGAACGCAGCGAAATGCGATACTTGGTGTGAATTGC

AGAATCCCGTGAACCATCGAGTCTTTGAACGCAAGTTGCGCCCGAGGCTATCCGGCCGAG

GGCACGCCTGCCTGGGCGTCACGCCTCGCGTCGCTCCGTGCACCCCGTCCCGATGAGGCG

GCGGGTGCGGATGCGGAGATTGGCCCCCCGTGCCTGACGGCGCGGCGGGTCGAAGTGCGT

ACCGCCGGTCGGGACGGACGCGGCGAGTGGTGGACGGACACGTACGGCGCTGAACGTCGC

CTCCGCCCCCCGGCCACGGCGGTACATGCAAGGAACCCACGCCGAGCATCCCTCGGAACA

CGACCCCAGGTCAG

>ITS_KF671258.1_Ophiopogon_j

CGAACTCGTAAACGCTCCTGCAGGGGCGGAGGGAGGGCGCGGATATTCCGACCGCCCGAT

CTCGGTACCGCGGGGCACCATGGCCGCCCCCGCCCCGCATTGCTGCGGGACGGGCGGCGG

GAACAACACCCGGCGCGATGGGCGCCAAGGAACAATGCTTTGTCGGAGAGCGTCGCGTGC

CGGTCTCGGCGCGCAGCGTGATCCTTCCATACGTCGAACCTTTACGACTCTCGGCAACGG

ATATCTTGGCTCTCGCATCGATGAAGAACGCAGCGAAATGCGATACTTGGTGTGAATTGC

AGAATCCCGTGAACCATCGAGTCTTTGAACGCAAGTTGCGCCCGAGGCTATCCGGCCGAG

GGCACGCCTGCCTGGGCGTCACGCCTCGCGTCGCTCCGTGCACCCCGTCCCGATGAGGCG

GCGGGTGCGGATGCGGAGATTGGCCCCCCGTGCCTGACGGCGCGGCGGGTCGAAGTGCGT

ACCGCCGGTCGGGACGGACGCGGCGAGTGGTGGACGGACACGTACGGCGCTGAACGTCGC

CTCCGCCCCCCGGCCACGGCGGTACATGCAAGGAACCCACGCCGAGCATCCCTCGGAACA

CGACCCCAGGTCAG

>ITS_KJ535720.1_Ophiopogon_j

CGAACTCGTAAACGCTCCTGCAGGGGCGGAGGGAGGGCGCGGATATTCCGACCGCCCGAT

CTCGGTACCGCGGGGCACCATGGCCGCCCCCGCCCCGCATTGCTGCGGGACGGGCGGCGG

GAACAACACCCGGCGCGATGGGCGCCAAGGAACAATGCTTTGTCGGAGAGCGTCGCGTGC

CGGTCTCGGCGCGCAGCGTGATCCTTCCATACGTCGAACCTTTACGACTCTCGGCAACGG

ATATCTTGGCTCTCGCATCGATGAAGAACGTAGCGAAATGCGATACTTGGTGTGAATTGC

AGAATCCCGTGAACCATCGAGTCTTTGAACGCAAGTTGCGCCCGAGGCTATCCGGCCGAG

GGCACGCCTGCCTGGGCGTCACGCCTCGCGTCGCTCCGTGCACCCCGTCCCGATGAGGCG

GCGGGTGCGGATGCGGAGATTGGCCCCCCGTGCCTGACGGCGCGGCGGGTCGAAGTGCGT

ACCGCCGGTCGGGACGGACGCGGCGAGTGGTGGACGGACACGTACGGCGCTGAACGTCGC

CTCCGCCCCCCGGCCACGGCGGTACATGCAAGGAACCCACGCCGAGCATCTCTCGGAACA

CGACCCCAGGTCAG

>ITS_KF671254.1_Ophiopogon_j

CGAACTCGTAAACGCTCCTGCAGGGGCGGAGGGAGGGCGCGGATATTCCGACCGCCCGAT

CTCGGTACCGCGGGGCACCATGGCCGCCCCCGCCCCGCATTGCTGCGGGACGGGCGGCGG

GAACAACACCCGGCGCGATGGGCGCCAAGGAACAATGCTTTGTCGGAGAGCGTCGCGTGC

CGGTCTTGGCGCGCAGCGTGATCCTTCCATACGTCGAACCTTTACGACTCTCGGCAACGG

ATATCTTGGCTCTCGCATCGATGAAGAACGCAGCGAAATGCGATACTTGGTGTGAATTGC

AGAATCCCGTGAACCATCGAGTCTTTGAACGCAAGTTGCGCCCGAGGCTATCCGGCCGAG

GGCACGCCTGCCTGGGCGTCACGCCTCGCGTCGCTCCGTGCACCCCGTCCCGATGAGGCG

GCGGGTGCGGATGCGGAGATTGGCCCCCCGTGCCTGACGGCGCGGCGGGTCGAAGTGCGT

ACCGCCGGTCGGGACGGACGCGGCGAGTGGTGGACGGACACGTACGGCGCTGAACGTCGC

CTCCGCCCCCCGGCCACGGCGGTACATGCAAGGAACCCACGCCGAGCATCCCTCGGAACA

CGACCCCAGGTCAG

>ITS_AB721393.1_Ophiopogon_j

CGAACTCGTAAACGCTCCTGCAGGGGCGGAGGGAGGGCGCGGATATTCCGACCGCCCGAT

CTCGGTACCGCGGGGCACCATGGCCGCCCCCGCCCCGCATTGCTGCGGGACGGGCGGCGG

GAACAACACCCGGCGCGATGGGCGCCAAGGAACAATGCTTTGTCGGAGAGCGTCGCGTGC

CGGTCTTGGCGCGCAGCGTGATCCTTCCATACGTCGAACCTTTACGACTCTCGGCAACGG

ATATCTTGGCTCTCGCATCGATGAAGAACGTAGCGAAATGCGATACTTGGTGTGAATTGC

AGAATCCCGTGAACCATCGAGTCTTTGAACGCAAGTTGCGCCCGAGGCTATCCGGCCGAG

GGCACGCCTGCCTGGGCGTCACGCCTCGCGTCGCTCCGTGCACCCCGTCCCGAKGAGGCG

GCGGGTGCGGATGCGGAGATTGGCCCCCCGTGCCTGACGGCGCGGCGGGTCGAAGTGCGT

ACCGCCGGTCGGGACGGACGCGGCGAGTGGTGGACGGACACGTACGGCGCTGAACGTCGC

CTCCGCCCCCCGGCCACGGCGGTACATGCAAGGAACCCACGCCGAGCATCCCTCGGAACA

CGACCCCAGGTCAG

>ITS_KC439470.1_Ophiopogon_j

CGAACTCGTAAACGCTCCTGCAGGGGCGGAGGGAGGGCGCGGATATTCCGACCGCCCGAT

CTCGGTACCGCGGGGCACCATGGCCGCCCCCGCCCCGCATTGGTGCGGGACGGGCGGCGG

GAACAACACCCGGCGCGATGGGCGCCAAGGAACAATGCTTTGTCGGAGAGCGTCGCGTGC

CGGTCTTGGCGCGCAGCGTGATCCTTCCATACGTCGAACCTTTACGACTCTCGGCAACGG

ATATCTTGGCTCTCGCATCGATGAAGAACGTAGCGAAATGCGATACTTGGTGTGAATTGC

AGAATCCCGTGAACCATCGAGTCTTTGAACGCAAGTTGCGCCCGAGGCTATCCGGCCGAG

GGCACGCCTGCCTGGGCGTCACGCCTCGCGTCGCTCCGTGCACCCCGTCCCGATGAGGCG

GCGGGTGCGGATGCGGAGATTGGCCCCCCGTGCCTGACGACGCGGCGGGTCGAAGTGCGT

ACCGCCGGTCGGGACGGACGCGGCGAGTGGTGGACGGACACGTACGGCGCTGAACGTCGC

CTCCGCCCCCCGGCCACGGCGGTACATGCAAGGAACCCACGCCGAGCATCCCTCGGAACA

CGACCCCAGGTCAG

>ITS_KF671251.1_Ophiopogon_j

CGAACTCGTAAACGCTCCTGCAGGGGCGGAGGGAGGGCGCGGATATTCCGACCGCCCGAT

CTCGGTACCGCGGGGCACCATGGCCGCCCCCGCCCCGCATTGCTGCGGGACGGGCGGCGG

GAACAACACCCGGCGCGATGGGCGCCAAGGAACAATGCTTTGTCGGAGAGCGTCGCGTGC

CGGTCTTGGCGCGCAGCGTGATCCTTCCATACGTCGAACCTTTACGACTCTCGGCAACGG

ATATCTTGGCTCTCGCATCGATGAAGAACGTAGCGAAATGCGATACTTGGTGTGAATTGC

AGAATCCCGTGAACCATCGAGTCTTTGAACGCAAGTTGCGCCCGAGGCTATCCGGCCGAG

GGCACGCCTGCCTGGGCGTCACGCCTCGCGTCGCTCCGTGCACCCCGTCCCGATGAGGCG

GCGGGTGCGGATGCGGAGATTGGCCCCCCGTGCCTGACGGCGCGGCGGGTCGAAGTGCGT

ACCGCCGGTCGGGACGGACGCGGCGAGTGGTGGACGGACACGTACGGCGCTGAACGTCGC

CTCCGCCCCCCGGCCACGGCGGTACATGCAAGGAACCCACGCCGAGCATCCCTCGGAACA

CGACCCCAGTCAG

>ITS_EU930854.1_Ophiopogon_j

CGAACTCGTAAACGCTCCTGCAGGGGCGGAGGGAGGGCGCGGATATTCCGACCGCCCGAT

CTCGGTACCGCGGGGCACCATGGCCGCCCCCGCCCCGCATTGCTGCGGGACGGGCGGCGG

GAACAACACCCGGCGCGATGGGCGCCAAGGAACAATGCTTTGTCGGAGAGCGTCGCGTGC

CGGTCTTGGCGCGCAGCGTGATCCTTCCATACGTCGAACCTTTACGACTCTCGGCAGCGG

ATATCTTGGCTCTCGCATCGATGAAGAACGTAGCGAAATGCGATACTTGGTGTGAATTGC

AGAATCCCGTGAACCAGCGAGTCTTTGAACGCAAGTTGCGCCCGAGGCTATCCGGCCGAG

GGCACGCCTGCCTGGGCGTCACGCCTCGCGTCGCTCCGTGCACCCCGTCCCGATGAGGCG

GCGGGTGCGGATGCGGAGATTGGCCCCCCGTGCCTGACGGCGCGGCGGGTCGTAGTGCGT

ACCGCCGGTCGGGACGGACGCGGCGAGTGGTGGACGGACACGTACGGCGCTGAACGTCGC

CTCCGCCCCCCGGCCACGGCGGTACATGCAAGGAACCCACGCCGAGCATCCCTCGGAACA

CGACCCCAGGTCAG
